# The Phytochemical Profiling, In Vitro Antioxidant, and Hepatoprotective Activity of *Prenanthes purpurea* L. and Caffeoylquinic Acids in Diclofenac-Induced Hepatotoxicity on HEP-G2 Cells

**DOI:** 10.3390/ijms241814148

**Published:** 2023-09-15

**Authors:** Rositsa Mihaylova, Reneta Gevrenova, Alexandra Stefanova, Dimitrina Zheleva-Dimitrova, Vessela Balabanova, Gökhan Zengin, Rumyana Simeonova, Georgi Momekov

**Affiliations:** 1 Department of Pharmacology, Pharmacotherapy and Toxicology, Faculty of Pharmacy, Medical University of Sofia, 1000 Sofia, Bulgaria; rmihaylova@pharmfac.mu-sofia.bg (R.M.); astefanova22@gmail.com (A.S.); rsimeonova@pharmfac.mu-sofia.bg (R.S.); 2 Department of Pharmacognosy, Faculty of Pharmacy, Medical University of Sofia, 1000 Sofia, Bulgaria; rgevrenova@pharmfac.mu-sofia.bg (R.G.); dzheleva@pharmfac.mu-sofia.bg (D.Z.-D.); vbalabanova@pharmfac.mu-sofia.bg (V.B.); 3 Physiology and Biochemistry Research Laboratory, Department of Biology, Science Faculty, Selcuk University, Konya 42130, Turkey; gokhanzengin@selcuk.edu.tr

**Keywords:** hepatoprotection, *Prenanthes parpurea*, polyphenols, chlorogenic acid, 3,5-DiCQA, antioxidants, NSAIDs, hepatotoxicity

## Abstract

Oxidative stress is a common phenomenon of many liver disorders; it both affects patient survival and directly influences the applicability, effectiveness, and toxicity of drugs. In the pursuit of reliable natural remedies for hepatoprotection, this study reports on the complete phytochemical characterization, antioxidant, and hepatoprotective activities of the *Prenanthes purpurea* methanol-aqueous extract in an in vitro model of diclofenac-induced liver injury (DILI). An ultra high-performance liquid chromatography–high-resolution mass spectrometry analysis (UHPLC-HRMS) was conducted, delineating more than 100 secondary metabolites for the first time in the species, including a series of phenolic acid-hexosides, acylquinic, acylhydroxyquinic and acyltartaric acids, and flavonoids. Quinic acid, chlorogenic, 3,5-dicaffeoylquinic and 5-feruloylhydroxyquinic acid, caffeoyltartaric and cichoric acids, eryodictiol-*O*-hexuronide, and luteolin *O*-hexuronide dominated the phytochemical profile and most likely contributed to the observed hepatoprotective activity of the studied *P. purpurea* leaf extract. The potency and molecular basis of cellular protection were investigated in parallel with pure caffeoylquinic acids in a series of pretreatment experiments that verified the antiapoptotic and antioxidant properties of the natural products.

## 1. Introduction

Hepatic injury and function have extensively been studied in numerous experimental and clinical settings. The toxicodynamic mechanisms of well-known liver-targeting noxious stimuli have successfully been recreated in various experimental models in the search for promising strategies to counteract them. Being the central detoxifying organ, the liver is exposed to the direct damaging action of virtually any xenobiotic; however, drug-induced hepatotoxicity is a hallmark side effect of a few pharmacological classes including non-steroidal anti-inflammatory drugs (NSAIDs), antibiotics, antituberculosis, and antiepileptic medications [1,2]. Furthermore, various infectious and other diseases or conditions (i.e., virus infections, obesity, alcohol abuse, hereditary and autoimmune disorders, etc.) have been implicated in liver failure, exacerbating the urgency for efficient therapeutic approaches for hepatoprotection.

Acute hepatitis is the most common form of drug-induced liver injury (DILI), which in 10% of cases, is linked to NSAID use [3,4]. On one hand, these are among the most commonly used medications available over the counter and by prescription; and on the other, they comprise a widely heterogenous group in terms of both structural and therapeutic properties [4,5]. Diclofenac, an acetic acid derivative, has been associated with a significantly higher incidence of hepatotoxicity than other NSAIDs, causing idiosyncratic DILI of a mostly hepatocellular (cytolytic) type [3,4,6,7]. The precise molecular mechanisms by which diclofenac impairs liver function are multifactorial and not thoroughly elucidated; however, several toxicological models have been proposed. A potential role in idiosyncratic hepatotoxicity is played by the generation of reactive quinone imine metabolites and ROS in the first phase of diclofenac’s metabolism and the subsequent impairment of mitochondrial function [8,9,10,11]. Undergoing drug glucuronidation in the conjugation phase facilitates the formation of glucuronide–protein adducts with neoantigenic properties, thus inducing an autoimmune response culminating in hepatocyte death [9,12,13]. In light of these findings, inducing the intrinsic antioxidant defense system of hepatocytes has been the most lucrative strategy for shielding the organ from oxidative stress damage, which is recognized as a primary pathogenetic factor in DILI, as well as other liver diseases.

In vitro models of diclofenac-induced toxicity on both isolated animal hepatocytes and immortalized hepatic cell lines have been extensively used to study the molecular basis of DILI and the possible mechanisms of liver protection and regeneration by pharmacological and natural remedies [7,14,15]. About 50% of the hepatoprotective products and additives used nowadays are of plant origin, many of which have undergone clinical trials targeting patient groups with different liver conditions, including silymarin, picroside, phyllanthin, glycyrrhizin, curcumin, and berberine, among others [14,16]. Many food and medicinal plants are evidenced to be abundant in polyphenolic phytochemicals with a prominent ROS scavenging capacity and other biologically active constituents with the ability to boost antioxidant enzyme activity and modulate gene expression and cell survival pathways [16,17]. 

Of the many classes of biologically active secondary metabolites, caffeic acid derivatives are receiving growing attention due to mounting evidence of their antioxidant and hepatoprotective activity [18,19]. Moreover, different sub-classes of these polyphenolic conjugates, such as caffeoyl quinic and caffeoyl tartaric acids, have been proven to be reliable chemophenetic markers within the plant species from the Cichorieae tribe of the Asteraceae family [20].

The genus *Prenanthes* L. within the subtribe Hypochaeridinae (Cichorieae tribe, Asteraceae family) is widely distributed in Asia, Africa, North America, and Central and Southern Europe and consists of around 30 species [21,22,23,24]. Regarding their chemical composition, the species of the subtribe are a rich source of various valued secondary metabolites, with the flavonoids and sesquiterpenoids being characteristic for the taxa [25,26,27]. The most widely and commonly researched metabolites have been caffeic acid derivatives isolated from economically important taxa, such as *Cichorium intybus*, *Lactuca sativa*, and *Taraxacum officinale* [28]. However, this is the first extensive study on the phytochemical profile and biological activity of the investigated Cichorieae species, namely *Prenanthes purpurea*. Previously, chlorogenic, 3,5-dicaffeoylquinic, caffeoyl tartaric, and cichoric acids were reported [24], and a new di-*O*-cinnamoyl-trisaccharide derivative was isolated from the methanolic extract of *P. purpurea* leaves (Austrian origin) [29]. From the polar fraction of the *P. acerifolia* water extract, a new guaiane-type sesquiterpene lactone, prenanthelide A, and three new guaiane-type sesquiterpene glycosides—prenanthesides A, B, and C—have been isolated together with three known guaiane-type sesquiterpene glycosides, ixerin D, 8-epidesacylcynaropicrin glucoside, and crepiside E [30]. As for their biological activity, the presence of antidiabetic, antibacterial, analgesic, and anti-inflammatory properties of oncocalyxone A isolated from *P. sarmentosus* leaves has been established [31]. 

Hence, as a part of our outgoing investigation on Asteraceae species, *P. purpurea* was subjected to a phytochemical and biological characterization. To the best of our knowledge and based on the above-mentioned literature survey, there are no profound inquiries on the in vitro antioxidant and hepatoprotective activity of the studied taxon. Additionally, in-depth studies on both the chemical composition and the biological activity of the species are missing and appear to be worth value.

The main objectives of this study were to provide a phytochemical profiling of the aerial parts of *Prenanthes purpurea* (PE) and to evaluate its hepatoprotective activity in a comparative manner to plant-derived polyphenols chlorogenic acid (CA) and 3,5-dicaffeoylquinic acid (3,5-DiCQA). The pharmacodynamic properties of PE and one of its major constituents, CA, were studied in the context of oxidative stress in a suitable in vitro model (HEP-G2 cells) of diclofenac-induced cytotoxicity, tracing expression changes in a cluster of related proteins. The antagonistic effect of PE, CA, and 3,5-DiCQA on NSAID drug toxicity was estimated in a detailed “dose-effect” analysis of each drug combination using Compusyn^®^ 1.0 software based on the Chou-Talalay method [32].

## 2. Results and Discussion

### 2.1. A Secondary Metabolite Profiling of a P. purpurea Extract

**Figure 1:**
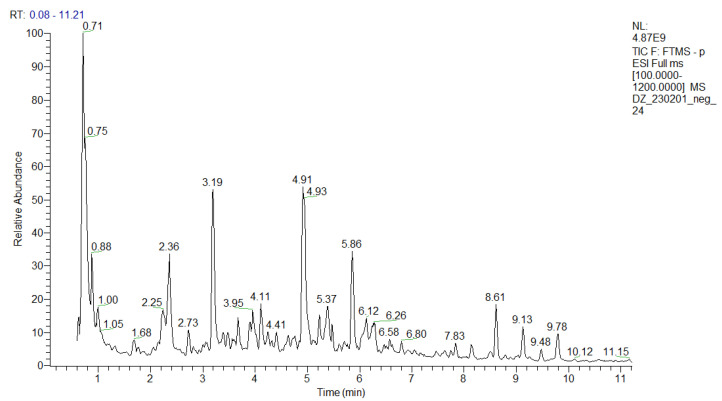
TIC in negative ion mode of the *P. purpurea* extract.

An in-depth UHPLC-HRMS analysis of a *P. purpurea* methanol-aqueous extract was performed, allowing for the identification/annotation of more than 100 secondary metabolites (Table 1 and Table S1). To the best of our knowledge, this study is the first attempt at a phytochemical profiling of the species. Herein, 39 compounds were assigned to phenolic acids and derivatives and simple coumarins including a series of hydroxybenzoic and hydroxicinnamic acids glycosides and 6 phenylethanoid glycosides. Thirty-one compounds were ascribed as acylquinic acids, among them 6 monoacylhydroxyquinic acids, 5 acylquinic acids hexosides, and 2 caffeoyl-hydroxydihydrocaffeoylquinic acid. Within a group of acyltartaric acids, *p*-coumaroyl-caffeoyltartaric, feruloyl-caffeoyltartaric, and dicaffeoyltartaric acids were tentatively identified. Among the flavonoids, it is worth noting the presence of hexuronides and the acetylhexosides of apigenin, luteolin, and naringenin. The total ion chromatogram (TIC) in negative ion mode of the studied extract was depicted in Figure 1.

This study is also the first attempt at an in-depth secondary metabolite annotation of the species from the subtribe Hypochaeridinae (Cichorieae tribe, Asteraceae family) using the hyphenated technique LC-MS, allowing for the identification of a series of acylquinic, acylhydroxyquinic, and acyltartaric acids and flavonoids not previously reported even in the subtribe taxa (Table 1 and Table S1, Figure 1 and Figures S1–S4).

#### 2.1.1. Hydroxybenzoic and Hydroxycinnamic Acids and Their Derivatives

**Figure 2:**
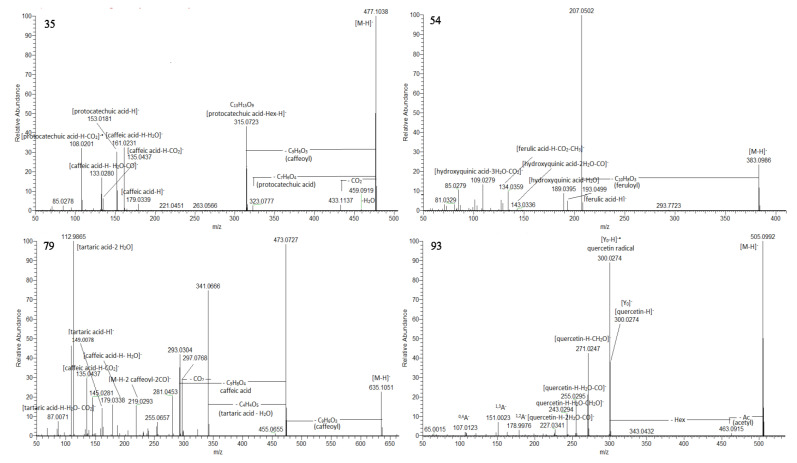
MS/MS spectra of protocatechuic acid –(caffeoyl)-hexoside 1 (**35**), 5-feruloyl-2-hydroxyquinic acid (**54**), tricaffeoyltartaric acid (**79**), and quercetin-acetylhexoside (**93**).

Numerous hydroxybenzoic and hydroxycinnamic acid glycosides were annotated including hexosides (**2**–**5**, **8**, **9**, **11**, **12**, **14**, **17**, **18**, **23** and **30**) together with a hexosylpentoside of protocatechuic acid (**10**) and deoxyhexoside of vanillic (**22**), and syringic acid (**28**) (Table 1). Compounds **35**, **36,** and **38** shared the same deprotonated molecules [M-H]^−^ at *m*/*z* 477.104 (calc. for C_22_H_21_O_12_). The presence of caffeoyl and protocatechuyl residue was deduced from the transitions 477.104→315.072 [M-H-C_9_H_6_O_3_]^−^ and 315.072→153.0181 [M-H- (C_9_H_6_O_3_ + C_6_H_10_O_5_]^−^, supported by the prominent fragment ions at *m*/*z* 161.023 [caffeic acid-H-H_2_O]^−^ and 153.018 [protocatechuic acid-H-H_2_O]^−^. Accordingly, **35**, **36,** and **38** were assigned to protocatechuic acid-(caffeoyl)-hexosides (Figure 2). In the same way, syringic acid at *m*/*z* 197.045, gallic acid at *m*/*z* 169.013, and hydroxybenzoic acid at *m*/*z* 137.023 was dedicated in **24**, **34,** and **39**, respectively. Thus, they were ascribed as syringic acid (caffeoyl)-hexoside, gallic acid-(caffeoyl)-hexoside, and caffeic acid-(hydroxybensoyl)-hexoside. The MS/MS spectra of sugar esters hydroxybensoyl-hexose (**7**) and caffeoyl-hexose (**13**) were acquired. The precursor ions provided fragment ions resulting from the hexose cross ring cleavages as ^0,4^Hex (−60 Da), ^0,3^Hex (−90Da), and ^0,2^Hex (−120 Da) [33]. Based on the comparison with the retention times and fragmentation patterns of reference standards, 5 hydroxybenzoic acids (**1**, **6**, **16**, **33** and **37**) and 4 hydroxycinnamic acids (**19**, **25**, **27** and **31**) together with umbelliferone (**20**) and quinic acid (**21**) were identified in the extracts (Table S1, Figure S1).

Within this group, **21** (quinic acid) (7.76%) was the main compound in the studied extract, together with **4** (6.07%), **7** (6.04%), **8** (6.01%), and **15** (5.20%) (Figure S1). Although hydroxybenzoic and hydroxycinnamic acids were present in their free forms, a large number of phenolic acids hexosides were evidenced herein in *P. purpurea* for the first time.

#### 2.1.2. Acylquinic Acids (AQAs)

Overall, 8 monoAQA and 10 diAQA acids together with 6 monoacylhydroxyquinic acids and 4 feruloylquinic acid glycosides and 1 dicaffeoylquinic acid hexoside were dereplicated or annotated in the *P. purpureum* extract (Table 1 and Table S1, Figure S2). The AQAs annotation was based on diagnostic ions and their relative abundances corresponding to each subclass AQAs [34,35,36]. Thus, the base peaks at *m*/*z* 191.055 indicated substitution at C-5 of the quinic acid skeleton. Accordingly, **43**/**51**, **47**/**56,** and **52/60** were ascribed as 5-caffeoyl-, 5-feruloyl-, and 5-p-coumaroylquinic acid and their isomers, respectively. 

The assignment of feruloylquinic acid-hexoside/pentoside (**41**/**46** and **48**/**59**) was suggested by the transitions 529.157→367.103 and 499.146→367.103, respectively, resulting from the losses of hexosyl (162.053 Da) and pentosyl units (132.043 Da) (Table S1). 

Compounds **42**/**49** ([M-H]^−^ at *m*/*z* 369.083), **45**/**58** ([M-H]^−^ at *m*/*z* 353.088), and **54**/**61** ([M-H]^−^ at *m*/*z* 383.099) provided a base peak at *m*/*z* 207.050 (calc. for C_7_H_11_O_7_), indicating hydroxyquinic acid (HQA) and losses of caffeoyl, *p*-coumaroyl and feruloyl residue, respectively (Table S1). Additionally, the presence of hydroxyquinic acid was supported by the series of prominent fragment ions at *m*/*z* 189.040 [HQA-H-H_2_O]^−^, 143.034 [HQA-H-2H_2_O-CO]^−^, 127.039 [HQA-H-2H_2_O-CO_2_]^−^, 109.028 [HQA-H-3H_2_O-CO_2_]^−^, and 85.028 [HQA-H-C_3_H_6_O_5_]^−^. Via analogy with monoAQAs, **42**/**49** were related to 5-caffeoylHQA, **45**/**58**—to 5-*p*-coumaroylHQA, while 5-feruloylHQA were deduced for **54**/**61** (Figure 2).

Compounds **50** and **55** shared the same [M-H]^−^ at *m*/*z* 677.194 (calc. for C_28_H_37_O_19_ (Table 1 and Table S1). They afforded indicative fragment ions at *m*/*z* 383.099 [M-H-C_11_H_18_O_9_]^−^, resulting from the concomitant losses of pentose and hexose unit. Hydroxyquinnic and feruloyl acid were evidenced by the prominent ions at *m*/*z* 207.050 [M-H-Pent-Hex-C_10_H_8_O_3_]^−^ and 193.050 [M-H-Pent-Hex-Ferulic acid]^−^, supported by the corresponding series of neutral and radical (from feruloyl residue) losses. Accordingly, **50** and **55** were annotated as isomeric 5-feruloyl-HQA- hexosylpentoside. 

**Table 1:** Secondary metabolites in *P. purpurea* methanol-aqueous extracts.

No.	Identified/Tentatively Annotated Compound	Molecular Formula	Exact Mass [M-H]^−^	t_R_ (min)	Δppm	Level of Confidence [37]
**Hydroxybenzoic and hydroxycinnamic acids, and phenylethanoid glycosides**						
**1**	gallic acid ^a^	C_7_H_6_O_5_	169.0142	1.15	−7.198	1
**2**	gallic acid-hexoside	C_13_H_15_O_10_	331.0678	1.22	0.212	2
**3**	hydroxybenzoic acid-*O*-hexoside	C_13_H_16_O_8_	299.0778	1.29	3.074	2
**4**	protocatechuic acid-*O*-hexoside	C_13_H_16_O_9_	315.0727	1.67	0.269	2
**5**	vanillic acid-*O*-hexoside	C_14_H_18_O_9_	329.0875	1.76	2.111	2
**6**	protocatechuic acid ^a^	C_7_H_6_O_4_	153.0181	2.04	−9.358	1
**7**	*O*-hydroxybenzoyl-hexose	C_13_H_16_O_8_	299.0778	2.06	−0.002	2
**8**	protocatechuic acid-*O*-hexoside	C_13_H_16_O_9_	315.0727	2.11	0.459	2
**9**	syringic acid-*O*-hexoside	C_15_H_20_O_10_	359.0985	2.27	0.808	2
**10**	protocatechuic acid-hexosylpentoside	C_18_H_24_O_13_	447.1144	2.37	0.394	2
**11**	caffeic acid *O-* hexoside	C_15_H_18_O_9_	341.0871	2.42	−4.677	2
**12**	hydroxybenzoic acid-*O*-hexoside	C_13_H_16_O_8_	299.0778	2.45	0.600	2
**13**	*O*-caffeoyl hexose	C_15_H_18_O_9_	341.0871	2.50	−2.537	2
**14**	gentisic acid-*O*-hexoside	C_13_H_16_O_9_	315.0727	2.58	1.316	2
**15**	aesculetin-*O*-hexoside	C_15_H_15_O_9_	339.0724	2.73	0.515	2
**16**	4-hydroxybenzoic acid	C_7_H_6_O_3_	137.0230	2.85	−10.709	2
**17**	hydroxybenzoic acid-*O*-hexoside	C_13_H_16_O_8_	299.0778	3.01	2.539	2
**18**	caffeic acid *O*-hexoside	C_15_H_18_O_9_	341.0871	3.08	−0.367	2
**19**	*p*-coumaric acid^a^	C_9_H_8_O_3_	163.0389	3.10	−7.835	1
**20**	umbelliferone	C_9_H_6_O_3_	161.0244	3.16	−8.057	2
**21**	quinic acid	C_7_H_12_O_6_	191.0549	3.19	−5.921	2
**22**	vanillic acid *O*-deoxyhexoside	C_14_H_18_O_8_	313.0929	3.26	0.509	2
**23**	coumaric acid-*O-*hexoside	C_15_H_18_O_8_	325.0930	3.33	−1.386	2
**24**	syringic acid-(caffeoyl)-pentoside	C_22_H_20_O_13_	491.0831	3.34	−0.028	2
**25**	*m-*coumaric acid ^a^	C_9_H_8_O_3_	163.0389	3.35	−8.203	1
**26**	aesculetin	C_9_H_6_O_4_	177.0193	3.46	−6.790	2
**27**	caffeic acid ^a^	C_9_H_8_O_4_	179.0339	3.55	−5.810	1
**28**	syringic acid *O*-deoxyhexoside	C_15_H_20_O_9_	343.1035	3.65	0.363	2
**29**	caffeoylmalic acid	C_13_H_12_O_8_	295.0459	4.18	0.676	2
**30**	coumaric acid-*O-*hexoside	C_15_H_18_O_8_	325.0930	4.46	0.490	2
**31**	*o*-coumaric acid ^a^	C_9_H_8_O_3_	163.0389	4.58	−8.326	1
**32**	caffeoylcitramalic acid	C_14_H_14_O_8_	309.0616	4.76	1.745	2
**33**	vanillic acid^a^	C_8_H_8_O_4_	167.0338	4.79	−7.376	1
**34**	gallic acid-(caffeoyl)-hexoside	C_22_H_22_O_13_	493.0988	5.18	0.661	2
**35**	protocatechuic acid-(caffeoyl)-hexoside 1	C_22_H_22_O_12_	477.1038	5.67	−0.124	2
**36**	protocatechuic acid-(caffeoyl)-hexoside 2	C_22_H_22_O_12_	477.1038	5.81	−0.900	2
**37**	salicylic acid ^a^	C_7_H_6_O_3_	137.0230	6.27	−10.928	1
**38**	protocatechuic acid-(caffeoyl)-hexoside	C_22_H_22_O_12_	477.1038	6.54	0.002	2
**39**	caffeic acid-(hydroxybensoyl)-hexoside	C_22_H_22_O_11_	461.1089	6.60	1.226	2
**Mono- and diacylquinic acids, and derivatives**						
**40**	neochlorogenic (3-caffeoylquinic) acid ^a^	C_16_H_18_O_9_	353.0867	2.36	0.410	1
**41**	4-feruloylquinic acid-hexoside	C_23_H_30_O_14_	529.1563	2.64	0.872	2
**42**	5-caffeoyl-2-hydroxyquinic acid	C_16_H_18_O_10_	369.0827	2.82	−0.379	2
**43**	chlorogenic (5-caffeoylquinic) acid ^a^	C_16_H_18_O_9_	353.0874	3.18	−1.403	1
**44**	4-caffeoylquinic acid	C_16_H_18_O_9_	353.0878	3.37	0.495	2
**45**	5-*p*-coumaroyl-2-hydroxyquinic acid 2	C_16_H_18_O_9_	353.0878	3.59	−0.581	2
**46**	5-feruloylquinic acid-hexoside	C_23_H_30_O_14_	529.1563	3.61	0.872	2
**47**	3-feruloylquinic acid	C_17_H_20_O_9_	367.1034	3.71	4.954	2
**48**	5-feruloylquinic acid-pentoside	C_22_H_28_O_13_	499.1457	3.73	1.414	2
**49**	5-caffeoyl-2-hydroxyquinic acid 2	C_16_H_18_O_10_	369.0827	2.82	−0.379	2
**50**	5-feruloyl-hydroxyquinic acid-hexosylpentoside	C_28_H_38_O_19_	677.1929	3.19	1.119	2
**51**	5-caffeoylquinic acid isomer	C_16_H_18_O_9_	353.0874	3.90	0.155	2
**52**	5-*p*-coumaroylquinic acid	C_16_H_18_O_8_	337.0928	3.98	0.473	2
**53**	3-caffeoyl-5-hydroxy-dihydrocaffeoylquinic acid	C_25_H_26_O_13_	533.1288	4.03	0.574	2
**54**	5-feruloyl-2-hydroxyquinic acid	C_17_H_20_O_10_	383.0986	4.11	0.600	2
**55**	5-feruloyl-hydroxyquinic acid- hexosylpentoside	C_28_H_38_O_19_	677.1929	4.14	3.11	2
**56**	5-feruloylquinic acid	C_17_H_20_O_9_	367.1034	4.41	−0.260	2
**57**	1-caffeoyl-3-hydroxy-dihydrocaffeoylquinic acid	C_25_H_26_O_13_	533.1288	4.45	0.540	2
**58**	5-p-coumaroyl-2-hydroxyquinic acid 2	C_16_H_18_O_9_	353.0878	4.50	−0.723	2
**59**	5-feruloylquinic acid-pentoside	C_22_H_28_O_13_	499.1457	4.51	0.573	2
**60**	5-*p*-coumaroylquinic acid isomer	C_16_H_18_O_8_	337.0928	4.63	−0.180	2
**61**	5-feruloyl-2-hydroxyquinic acid	C_17_H_20_O_10_	383.0985	4.76	0.600	2
**62**	1, 3-dicaffeoylquinic acid-hexoside	C_31_H_34_O_17_	677.1723	5.16	1.325	2
**63**	3,4-dicaffeoylquinic acid ^a^	C_25_H_24_O_12_	515.1190	5.70	0.254	1
**64**	3,5-dicaffeoylquinic acid ^a^	C_25_H_24_O_12_	515.1189	5.85	−1.416	1
**65**	1,5-dicaffeoylquinic acid ^a^	C_25_H_24_O_12_	515.1190	6.02	0.720	1
**66**	4,5-dicaffeoylquinic acid	C_25_H_24_O_12_	515.1190	6.23	0.196	2
**67**	3-*p*-coumaroyl-5-caffeoylquinic acid	C_25_H_24_O_11_	499.1251	6.52	−0.210	2
**68**	3-caffeoyl-5-*p*-coumaroylquinic acid	C_25_H_24_O_11_	499.1251	6.58	−0.150	2
**69**	1-*p*-coumaroyl-5-caffeoylquinic acid	C_25_H_24_O_11_	499.1251	6.79	−0.751	2
**70**	4-caffeoyl-5-*p*-coumaroylquinic acid	C_25_H_24_O_11_	499.1252	6.98	−0.210	2
**Acyltartaric acids and derivatives**						
**71**	caffeoyltartaric acid 1	C_13_H_12_O_9_	311.0409	2.16	1.495	2
**72**	caffeoyltartaric acid 2	C_13_H_12_O_9_	311.0409	2.36	1.495	2
**73**	*p*-coumaroyltartaric caid	C_13_H_12_O_8_	295.0459	3.10	0.676	2
**74**	dicaffeoyltartaric acid-hexoside	C_28_H_28_O_17_	635.1254	4.32	−2.318	2
**75**	caffeoyl-dihydrocaffeoyltartaric acid	C_22_H_20_O_12_	475.0882	4.83	2.359	2
**76**	cichoric acid 1	C_22_H_18_O_12_	473.0725	4.91	−0.949	2
**77**	cichoric acid 2	C_22_H_18_O_12_	473.0725	5.22	−0.590	2
**78**	*p*-coumaroyl-caffeoltartaric acid 1	C_22_H_18_O_11_	457.0776	5.72	2.069	2
**79**	tricaffeoyltartaric acid	C_31_H_24_O_15_	635.1042	5.99	1.349	2
**80**	caffeoyl-feruloyltartaric acid 1	C_23_H_20_O_12_	487.0882	6.05	−2.647	2
**81**	*p*-coumaroyl-caffeoltartaric acid 2	C_22_H_18_O_11_	457.0776	6.10	−3.007	2
**82**	*p*-coumaroyl-caffeoltartaric acid 3	C_22_H_18_O_11_	457.0776	6.65	−17.034	2
**83**	caffeoyl-feruloyltartaric acid 2	C_23_H_20_O_12_	487.0882	7.87	0.926	2
**Flavonoids**						
**84**	luteolin *O-*hexosyl-(1→6)-hexoside(gentiobioside)	C_27_H_30_O_16_	609.1464	4.71	0.414	2
**85**	luteolin *O*-hexosyl-*O*-hexuronide	C_27_H_28_O_17_	623.1254	4.84	0.766	2
**86**	luteolin *O*-pentosyl-(1→2)-hexoside	C_26_H_28_O_15_	579.1355	5.15	0.547	2
**87**	isorhamnetin *O*-pentosyl-(1→2)-hexoside	C_27_H_30_O_16_	609.1464	5.16	0.414	2
**88**	luteolin 7-*O*-rutinoside ^a^	C_27_H_30_O_15_	593.1512	5.22	0.399	1
**89**	apigenin *O*-hexosyl-(1→6)-hexoside(gentiobioside)	C_27_H_30_O_15_	593.1512	5.30	0.500	2
**90**	eryodictiol *O*-hexuronide	C_21_H_20_O_12_	463.0882	5.34	0.218	2
**91**	luteolin-*O*-hexuronide	C_21_H_18_O_12_	461.0736	5.37	0.306	2
**92**	luteolin 7-*O*-glucoside ^a^	C_21_H_19_O_11_	447.0934	5.41	0.079	1
**93**	quercetin *O*-acetylhexoside	C_23_H_22_O_13_	505.0988	5.62	0.824	2
**94**	isorhamnetin 3-*O*-glucoside ^a^	C_22_H_21_O_12_	477.1044	6.02	2.056	1
**95**	apigenin 7-*O*-glucoside ^a^	C_21_H_19_O_10_	431.0980	6.08	−0.881	1
**96**	apigenin-*O*-hexuronide	C_21_H_18_O_11_	445.0774	6.12	−0.617	2
**97**	naringenin *O*-hexuronide	C_21_H_20_O_11_	447.0933	6.16	−1.755	2
**98**	luteolin *O*-malonylhexoside	C_24_H_22_O_14_	533.0939	6.18	0.472	2
**99**	luteolin *O*-acetylhexoside	C_23_H_22_O_12_	489.1038	6.19	0.101	2
**100**	kaempferol *O*-acetylhexoside	C_23_H_22_O_12_	489.1038	6.28	0.370	2
**101**	luteolin *O*-hexoside	C_21_H_19_O_11_	447.0934	6.41	0.079	2
**102**	naringenin O-acetylhexoside	C_23_H_24_O_11_	475.1246	6.89	3.505	2
**103**	luteolin ^a^	C_15_H_9_O_7_	285.0406	7.58	0.346	1

^a^—identified by comparison with an authentic standard; level of confidence: 1—compound identified by comparison to the reference standard; 2—putatively annotated compound.

DiAQA belong to a wide spread of Asteraceae subclasses: dicaffeoylquinic acids (diCQA) (**63**–**66**), p-coumaroyl-caffeoylquinic acids (*p*-CoCQA) (**67**–**70**), and hydroxydihydrocaffeoyl-caffeolylquinic acids (HC-CQA) (**53** and **57**). 

Compounds **53** and **68** provided diagnostic ions at *m*/*z* 353.088, indicating a loss of hydroxydihydrocaffeoyl (**53**) and *p*-coumaroyl (**68**) residue before caffeoyl moiety. Moreover, both compounds provided base peaks at *m*/*z* 191.055 accompanied by the abundant ion at *m*/*z* 179.034 [caffeic acid-H]^−^, as was observed in 3-CQA (Table 1 and Table S1). Thus, **53** and **68** were assigned to 3C-5HCQA and 3C-5-*p*-CoQA, respectively. 

Vicinal diCQA 3, 4-diCQA (**63**), 4,5-diCQA (**66**), and 4C-5-p-CoQA (**70**) were evidenced by the distinctive “dehydrated” ion of the quinic acid at *m*/*z* 173.045 (Table S1).

3, 5-diCQA (**64**) and 3-*p*-Co-5CQA (**67**) were deduced from the abundant fragment ions at *m*/*z* 353.088 [M-H-caffeoyl]^−^ and 337.093, respectively. Prominent ions at *m*/*z* 191.055 (79.6%) (**64**) and 163.039 (100%) (**67**) were produced as was seen in 3-CQA and 3-*p*-CoQA, respectively (Table 1 and Table S1).

Peak **62** yielded a precursor ion at *m*/*z* 677.173 (calc. for C_31_H_33_O_17_), along with the transitions at *m*/*z* 677.173→515.141→353.088→191.055 resulting from the losses of two caffeoyl residues and hexose unit, respectively (Table 1 and Table S1). A 1,3-disubstituted quinic acid skeleton was deduced from the fragment ions at *m*/*z* 191.055 (76%), 179.034 (95%), and 135.044 (100%). Thus, **62** was assigned to 1,3-dicaffeoylquinic acid-hexoside. Based on the comparison of retention times and fragmentation patterns of reference standards, compounds **40**, **43**, **63**–**65** were identified as neochlorogenic, chlorogenic, 3, 4-, 3, 5- and 1, 5-dicaffeoylquinic acid. The extracted ion chromatograms of acylquinic acids and derivatives showed that the *P. purpurea* profile was dominated by chlorogenic acid (**43**) (15.44%), 3,4-dicaffeoylquinic acid (**63**) (14.74%), 3,5-dicaffeoylquinic acid (**64**) (14.37%), and 1,5-dicaffeoylquinic acid (**65**) (13.00%), and 5-feruloyl-2-hydroxyquinic acid (**54**) (7.13%) (Figure S2).

#### 2.1.3. Acyltartaric Acids

A variety of acyltartaric acids (ATA) was annotated, including 2 monoATA, 8 diATA, 1 diATA-hexoside, and 1 triATA (Table 1 and Table S1, Figure 2 and Figure S3). Based on the distinctive fragment ion at *m*/*z* 149.008 [TA-H]^−^ (tartaric acid, TA) supported by the fragments at *m*/*z* 112.986 [TA-H-2H_2_O]^−^ and 103.002 [TA-H-H_2_O-CO]^−^, isomeric caffeoyltartaric acids **71** and **72**, and *p*-coumaroyltartaric acid (**73**) were annotated. Compounds **76** and **77** refer to dicaffeyltartaric (cichoric) acids yielding prominent fragment ions at *m*/*z* 311.041 (up to 84.2%) and 149.008 (100%). The assignment of three isobars of *p*-coumaroyl-caffeoyltartaric acid **78**, **81,** and **82** (at *m*/*z* 457.078, calc. for C_22_H_17_O_11_) was confirmed by the fragments at *m*/*z* 295.046 [M-H-caffeoyl]^−^ and 219.029 [M-H-coumaric acid-2CO]^−^ together with the abundant ions at *m*/*z* 179.033 [caffeic acid-H]^−^ and 163.034 [*p*-coumaric acid-H]^−^ (Table 1 and Table S1). In the same way, isobaric caffeoyl-feruloyltartaric acids **80** and **83** were deduced from the fragment ions at *m*/*z* 325.057 [M-H-caffeoyl]^−^ and 193.050 [ferulic acid-H]^−^ along with 134.036 [ferulic acid-H-CO_2_-CH_3_]^−^. ATAs show a high degree of stereoisomerism [38], as was observed in the *Cicerbita alpina* metabolite profiling [39]. Among acyltartaric acids, the predominant compounds in the *P. purpurea* extract were caffeoyl-dihydrocaffeoyltartaric acid (**75**) (32.92%), cichoric acid 1 (**76**) (32.83%), followed by caffeoyltartaric acid 2 (**72**) (14.86%), and cichoric acid 2 (**77**) (10.49%) (Figure S3).

#### 2.1.4. Flavonoids

In general, 12 flavone-, 4-flavonol-, and 3 flavanone-glycosides were assigned to flavonoids. The flavonoid annotation/dereplication was based on the diagnostic ions and their relative abundances corresponding to flavonols, flavones, and flavanones [34,35,36].

The sugar chain of **86** and **87** was consistent with pentosylhexoside (294 Da, C_16_H_18_O_9_) (Table S1). As an example, in (−) ESI-MS/MS the precursor ions at *m*/*z* 609.146 (**87**) yielded the deprotonated molecule of isorhamnetin (Y_0_^−^) at *m*/*z* 315.050 together with an abundant radical aglycone [Y_0_-H]^−•^ at *m*/*z* 314.043 (78.7%), thus suggesting a 3-*O*-glycosidic bond [40]. 

The aglycone was discernible by a prominent fragment at *m*/*z* 299.1097 [Y_0_-H-CH_3_]^−•^ and RDA ions at *m*/*z* 178.998 (^1,2^A^−^), 151.002 (^1,3^A^−^), and 107.012 (^0,4^A^−^). In (+) ESI, [M + H]^+^ at *m*/*z* 611.156 exhibited consecutive losses of a pentosyl moiety at *m*/*z* 479.118 (12.6%) and a hexosyl one at *m*/*z* 317.065 (100%). The assignment of the disaccharide was based on the fragmentation rules of Cuyckens and al., 2004 [40], where the abundances of both agycone and terminal monosaccharide favored pentosyl (1→2)-hexosyl interglycosidic linkage (Table S1). Concerning **84** ([M + H]^+^ at *m*/*z* 611.159) and **89** ([M + H]^+^ at *m*/*z* 595.165), hexosyl-(1→6)-hexoside was deuced from the dihexoside breakdown fragments at *m*/*z* 449.107 (25.2%) [M + H-Hex]^+^ and 433.112 (23.7%), respectively. Luteolin (**84**) and apigenin (**89**) were evidenced by the base peaks and RDA ions at *m*/*z* 151.002 (^1,3^A^−^) and 107.012 (^0,4^A^−^), 133.028 (^1,3^B^−^) (**84**), and 117.033 (^1,3^B^−^) (**89**).

A MS/MS spectra of **85** with [M-H]^−^ at *m*/*z* 623.126 and [M + H]^+^ at *m*/*z* 625.139 were acquired (Table S1). Prominent fragment ions at *m*/*z* 461.073 [M-H-Hex]^−^ and 449.106 [M + H-HexA]^+^ indicated both *O*-hexosyl and *O*-hexuronyl residue. Accordingly, **85** was ascribed as luteolin *O*-hexosyl-*O*-hexuronide.

Compounds **91**, **96** and **97** showed similar fragmentation patterns affording the base peaks at *m*/*z* 285.040 (**91**), 269.046 (**96**) and 271.061 (**97**) [M-H-HexA]^−^, respectively, suggesting flavonoid hexuronides. The aforementioned were consistent with luteolin-, apigenin-, and naringenin-*O*-hexuronide, respectively. 

Compounds **93**, **99**, **100,** and **102** were closely associated with the same fragmentation pattern yielding fragment ions indicating losses of the acetyl (−42 Da) and acetylhexosyl moieties (−204 Da) (Table S1). Accordingly, the aforementioned compounds were assigned to quercetin-, luteolin-, kaempferol-, and naringenin-*O*-acetylhexoside (Figure 2). 

One malonyl ester of luteolin-hexoside (**98**) was evidenced on the base of the loss of 86 Da (C_3_H_2_O_3_) at *m*/*z* 447.094 and 248 Da (C_9_H_12_O_8_) at *m*/*z* 285.040 (Table S1). 

Luteolin 7-*O*-glucoside (**92**), isorhamnetin 3-*O*-glucoside (**94**), and apigenin 7-*O*-glucoside (**95**) were unambiguously identified by comparison with reference standards.

Despite the fact that scutellarein (6-hydroxyapigenin) was reported in the subtribe Hypochaeridinae (Hedypnois, Leontodon) [41], the flavone was not proved herein.

The extracted ion chromatograms of flavonoids showed that the *P. purpurea* profile was dominated by eryodictiol-*O*-hexuronide (**90**) (22.48%), luteolin-*O*-hexuronide (**91**) (15.74%), together with apigenin-*O*-hexuronide (**96**) (13.12%), and naringenin *O*-hexuronide (**30**) (13.29%) (Figure S4). 

### 2.2. The Total Polyphenols, Flavonoid Contents, and Antioxidant Activity of the P. purpurea Extract

In this study, the contents of total polyphenols (TP) and total flavonoids (TF), as well as the antioxidant potential of the *P. purpurea* extract were evaluated using a variety of methods (Table 2). The *P. purpurea* extract demonstrated moderate levels of polyphenols and flavonoids. DPPH and ABTS+ were used to evaluate its radical scavenging capacity, while reduction abilities were calculated by the CUPRAC, FRAP, and phosphomolybdenum (PHMD) methods. The metal chelating method was based on the binding of transition metals by phytochemicals. Results are presented as trolox equivalents and ethylenediaminetetraacetic acid (EDTA), and the *P. purpurea* extract revealed the high activity of all of the used antioxidant methods. 

In line with the identified compounds and the received data for the total polyphenols, flavonoids content, and antioxidant activity of *P. purpurea* extract, the results confirm the significant correlation between the total polyphenol content and the presence of dihydroxycinnamic derivatives and caffeic acid derivatives as caffeoylquinic and caffeoyl tartaric acid and the DPPH-scavenging ability of the medicinal plant [42].

Oxidative stress is a key pathophysiological mechanism in many forms of liver diseases. Nevertheless, the application of antioxidants from various natural sources reveals its important clinical potential. In line with the above-mentioned, some of the secondary metabolites identified/annotated in *P. purpurea* aerial parts are recognized in the literature for their various properties in oxidative stress prevention. Protocatechuic and syringic acid have been noted for their potential antioxidant activity [43,44]. Furthermore, a number of experiments have confirmed the efficacy of vanillic and protocatechuic acid in the prevention of diabetes and neurodegenerative diseases, including Alzheimer’s [45,46]. Moreover, *p*-coumaric acid is well-known for its antioxidant capacity, prevention, and enhancement of diabetes and neuroprotection [47].

The presence of 3,5- and 4,5-diCQA, and chlorogenic acid suggests a strong antioxidant effect [48]. Additionally, quinic acid, another major compound in *P. purpurea* aerial parts, revealed prominent antioxidant activity, e.g., in the cell model of H_2_O_2_-induced oxidative stress [49]. It is worth noting that the presence of complex compounds carrying caffeoyl residue, such as chicoric and other caffeoyltartaric acids, contributed to the observed antioxidant and hepatoprotective effects of the *P. purpurea* extract. Moreover, the occurrence of luteolin, apigenin, and their glycosides suggests that the studied plant demonstrates considerable potential as a natural adjuvant treatment for inflammatory and oxidative stress diseases [50].

### 2.3. In Vitro Evaluation of the Hepatoprotective Activity 

#### 2.3.1. Results from the Cell Viability Assays

The in vitro hepatoprotective activity of a methanolic extract from the aerial parts of *P. purpurea* extract and the natural polyphenolic compounds chlorogenic acid (CA) and 3,5-DiCQA was first assessed in an in vitro model of diclofenac-induced cytotoxicity and oxidative damage in HEP-G2 cells. The design of the study aimed to compare the damaging capacity of a 24 h exposure to six different concentrations of diclofenac in unprotected HEP-G2 cells over cell cultures preincubated for 24 h with either the extract or the isolated phytoconstituents at a fixed 4:1 ratio.

**Figure 3:**
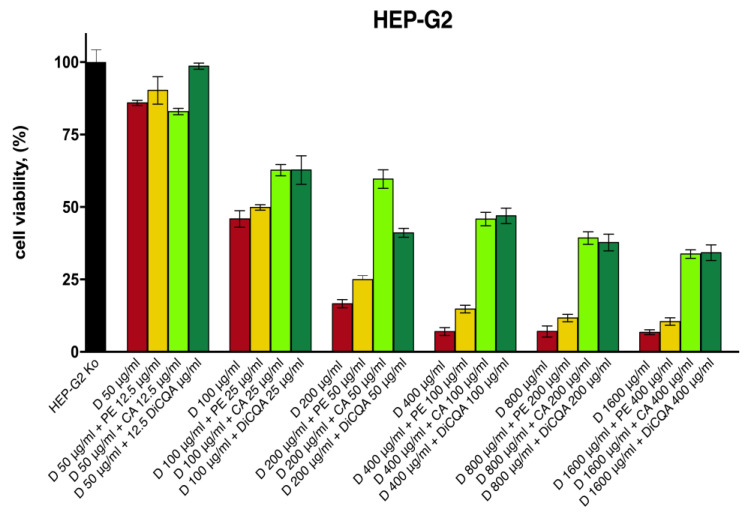
The cell viability of HEP-G2 cells following 24 h exposure to various concentrations of diclofenac: unprotected control group (red), pretreated for 24 h with serial dilutions of the *P. purpure*a extract (yellow), chlorogenic acid (light green), and DiCQA (dark green) in a fixed 4:1 dose ratio.

The results obtained in the cytotoxicity study indicate a positive modulating effect on diclofenac’s toxicity across all samples of the tested pretreatment combinations (Figure 3). As anticipated, the pure polyphenolic compounds CA and 3,5-DiCQA provided superior protection for hepatocyte survival, which was more pronounced in the intermediate and higher concentration data sets. Thereby, the percentage of viable cells was increased by several folds as compared with the unprotected diclofenac-treated groups, while the hepatoprotective activity of the isolated substances gradually weakened in the last two serial dilutions. The *Prenanthes* extract, on the other hand, exerted a more modest, yet consistent cell preserving effect that was less concentration dependent. The strongest hepatoprotective activity upon its 4:1 pretreatment combination was observed in the HEP-G2 samples exposed to 200 µg/mL and 400 µg/mL diclofenac, where cell survival was induced by ca. 50% and 100%, respectively. 

The counter aspect of the cell viability studies was to assess the effect of pretreatment on the cytotoxic potential of diclofenac against the HEP-G2 cell line (Table 3). A 24 h preincubation of hepatocyte cultures with the polyphenolic derivatives CA and 3,5-DiCQA resulted in a dramatic nearly two-fold increase in the half-inhibitory concentration of the NSAID (IC_50_ = 90.0 µg/mL estimated in the monotreatment regimen of diclofenac, as opposed to 174.1 and 160.3 µg/mL in its CA and DiCQA combinations, respectively). On the other hand, the PE pre-exposition had a milder mitigating effect on diclofenac’s cytotoxicity, escalating its equi-effective concentration by only ca. 10%.

#### 2.3.2. Results from the Compusyn^®^ Analysis

The Chou-Talalay methodology is a gold standard approach used to perform interaction analysis in drug combination studies, normally seeking to establish and quantitatively evaluate their synergistic behavior. It is, however, equally appropriate to use the same algorithm in characterizing combined drug effects with an opposite antagonistic pattern, as is essentially the case with all protective experimental studies [32]. 

In view of this, we subjected the MTT derived cytotoxicity data to an automated analysis in the Compusyn^®^ software, which estimated two major parameters CI (combination index) and DRI (drug reduction index) for the actual experimental as well as simulated data points of the “dose-response” curves. Thereby, the CI values provide a quantitative determination of synergistic (CI < 1), additive (CI = 1), and antagonistic (CI > 1) drug behavior in the fixed 4:1 (diclofenac: pretreatment agent) ratio combinations. Complementarily, DRIs (dose reduction indices) are plotted against the different fractions affected (Fa, % of unviable cells) and indicate the fold-change in the equi-effective concentrations of the single NSAID drug when used in combination for each dataset of the “dose-response” curves. When studying the combined effect of two drugs with an opposite in their nature action (i.e., cytotoxicity, cytoprotection), the stronger the antagonism, the higher the CI values for the inhibitory drug and the closer to 0 its DRI indices. 

In our study, the generated Compusyn^®^ reports yielded a full quantitative evaluation on the PE, CA, and DiCQA preconditioning effect on diclofenac’s toxicity all along the simulated “dose-response” curves. However, for the sake of simplicity and comprehensibility, we focused on interpreting the values of the two CI and DRI parameters calculated at the six experimental points (actual treatment concentrations).

Effect of PE pretreatment on diclofenac’s toxicity.

**Figure 4:**
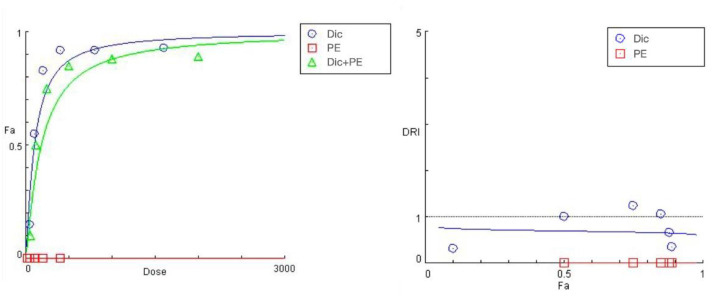
“Dose-response” curves (**left**) and DRI Fa plot (**right**) for the experimental data points of the studied combination PE + diclofenac.

The summarized data of the studied PE + diclofenac combination are presented in Figure 4 and Table 4. A 24 h pretreatment of HEP-G2 cells with the *Prenanthes* extract caused a right shift in diclofenac’s “dose-response” curve towards higher concentrations needed to produce the same effect on cell growth inhibition (Fa). According to the DRI-Fa plot, three out of the six DRI values fall in the 0–1 interval (implying antagonism and shown in bold in Table 3), covering both ends of the concentration range. The strongest modulating effect on diclofenac’s cytotoxicity was namely observed at the highest (1600:400) and lowest (50:12.5) exposure concentrations, where the DRI indices (0.35442 and 0.32125, respectively) indicated an approximately three-fold increase in the equi-inhibitory concentrations of the NSAID drug. The corresponding combination indices of CI at each experimental data set are infinitely high.

Effect of CA and 3,5-DiCQA pretreatment on Diclofenac’s toxicity.

**Figure 5:**
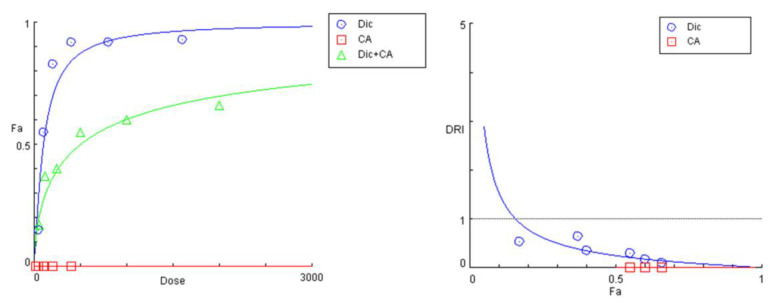
“Dose-response” curves and DRI-Fa plot for the experimental data points of the studied combination CA + diclofenac.

**Figure 6:**
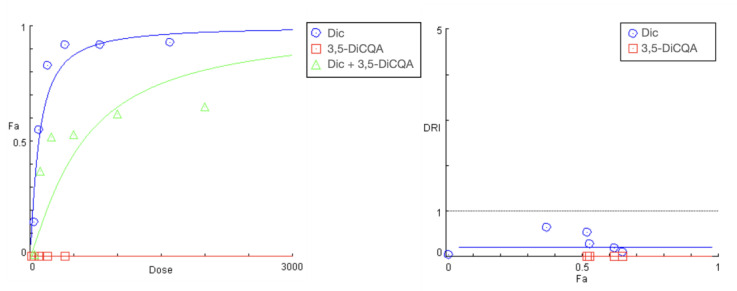
“Dose-response” curves and DRI-Fa plot for the experimental data points of the studied combination 3,5-DiCQA + diclofenac.

The preincubation of cell cultures with the two polyphenolic compounds CA and 3,5-DiCQA resulted in a similar outcome. However, there was a more pronounced rightward shift and a significant decrease in the slope of the “dose-response” curves of diclofenac in the tested combinations (Figure 5 and Figure 6). Both phytochemicals showed nearly matching profiles of hepatoprotection, which appears to be most pronounced under extreme hepatic cell injury (highest exposure doses to diclofenac) and gradually decreases with the lowering of the treatment concentrations (Table 5 and Table 6). The strongest antagonistic effect was established at the peak concentration pairs (1600:400 and 800:200 µg/mL), where estimated DRI indices point to, respectively, a nearly 10- and 5-fold reduction in the cytotoxic capacity of diclofenac. Similar to the PE combination study, the CI values at all actual data points were infinitely high.

#### 2.3.3. Results from the Proteome Profiling

**Figure 7:**
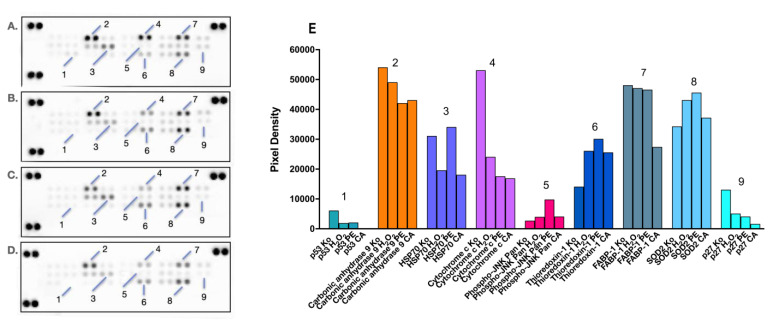
Changes in the expression levels of oxidative cell stress-related proteins in HEP-G2 cells exposed for 24 h to diclofenac in an unprotected control sample (**B**), PA (**С**), and CA (**D**) pretreatment regimens, as compared to the untreated control sample (**A**). Combination treatments were conducted in the same 4:1 dose ratio (400 diclofenac:100 µg/mL phytoprotectant), with the phytoprotection preceding the noxious stimuli by 24 h. After a total 48h incubation, a human proteome profiler immunoassay was performed according to the manufacturer’s instructions. Further densitometric analysis of the array spots was conducted using ImageJ software 1.8.0, and the most prominent changes in the proteome were expressed graphically (**E**). Legend: 1—p53; 2—Carbonic anhydrase 9; 3—HSP70; 4—Cytochrome c; 5—Phospho-JNK Pan; 6—Thioredoxin-1; 7—FABP-1; 8—SOD2; 9—p27.

The oxidative stress and antioxidant status are important aspects of any hepatic injury, including DILI. Considering this, we deemed it relevant to track and compare changes in cellular stress responses to diclofenac-induced damage in HEP-G2 cells and their coping mechanisms when pretreated with either PE (80% methanolic extract of the aerial parts of *Prenanthes purpurea*) or one of its major constituents, the polyphenolic compound chlorogenic acid (CA). Changes in the expression levels of multiple target proteins related to oxidative stress were monitored using proteome profiling in a series of immunoassays (Figure 7).

An important facet of cellular protection is the reversal of proapoptotic signaling of programmed cell death that occurred in both monotreated and pretreated samples, as evidenced by the decreased levels of the major tumor suppressors p53 (1) and p27 (9). The inhibition of these key cell cycle regulators is slightly more pronounced in the PE and CA pretreated group, where the 24 h CA preincubation resulted in the complete disappearance of their signaling spots. Furthermore, both the *Prenanthes* extract and CA prompted a nearly 3-fold reduction in the cytosolic levels of cytochrome C (4), which is a major recruiter and activator of procaspase-9 in the intrinsic mitochondrial pathway of apoptosis. Both pretreated cellular models also responded with a moderate downregulation of Carbonic anhydrase 9 (2), a major component in the HIF-mediated response and a cellular biomarker of hypoxia, which is almost exclusively expressed in solid tumors due to hypoxic conditions.

Changes in the expression levels of several factors with dual importance for both cell survival and redox homeostasis were also observed. For example, the preincubation of HEP-G2 cells with PE prior to diclofenac treatment produced a ca. 3-fold increase in the phosphorylated active form of all Janus kinases (Phospho-JNK Pan, (2)). JNK signaling has been recognized as central not only in cell survival and proliferation but also in promoting tolerance to oxidative stress and preventing ROS accumulation. Furthermore, the JAK/STAT pathway may promote the expression of another key survival factor, chaperone 70 (HSP70, (3)), which is also involved in cellular adaptation to oxidative stress. The observed expression profile of the latter protein was in good correlation with these findings, whereas its upregulation was favored only in the PE pretreated group. Significant reduction in the endogenous cytoprotectant liver-type fatty acid binding protein (FABP-1, (7)) was particularly seen in the CA pretreated sample, possibly due to its partial depletion in the course of antioxidative defense reactions, given its role in neutralizing free radicals. 

Finally, and most importantly, favorable changes were also observed in the availability of two pivotal factors directly involved in cellular detoxification, namely thioredoxin-1 (6) and superoxide dismutase 2 (SOD2, (8)). Thioredoxin is a highly conserved and ubiquitously expressed disulphide reductase and a major component of the redox system, functioning as an active scavenger of free radicals. SOD2, a member of the iron/manganese superoxide dismutase family, also played a central role in mitigating cellular stress by converting toxic superoxides to hydrogen peroxide. A marked induction in the expression levels of these proteins was established in all treatment groups; however, the strongest signals of their spots were detected in PE pretreated hepatocytes. 

Based on the conducted analysis of the proteomic imprints in both pretreated HEP-G2 groups, a general trend in their cellular responses can be derived, where CA is slightly more efficient in abrogating proapoptotic signaling, whereas the *Prenanthes* extract more directly affects cellular antioxidant machinery.

## 3. Materials and Methods

### 3.1. Plant Material

*P. purpurea* plant material (aerial parts) was collected at the Zlatnite Mostove locality, Vitosha Mt., Bulgaria at 1404 m a.s.l. (42.41° N 23.23° E) at full flowering stage, July 2022. The plant was identified by one of us (R.G) according to Stojanov et al. (1967) [23]. A voucher specimen was deposited at the Herbarium Academiae Scientiarum Bulgariae (SOM 177 803). The plant material was dried at room temperature.

### 3.2. Sample Extraction

Air-dried aerial parts (100 g) were extracted twice with 80% MeOH (1:20 *w*/*v*) by sonication (80 kHz, ultra-sound bath Biobase UC-20C) for 15 min at room temperature. The extracts were concentrated in vacuo and subsequently lyophilized (lyophilizеr Biobase BK-FD10P) to yield crude extracts of 8.125 g.

### 3.3. Chemicals

Acetonitrile (hypergrade for LC–MS), formic acid (for LC–MS) and methanol (an-alytical grade) were purchased from Merck (Merck, Bulgaria). The reference standards used for compound identification were obtained from Extrasynthese (Genay, France) for gallic, protocatechuic, *p*-coumaric, *m*-coumaric, *o*-coumaric, vanillic, and salicylic acid, as well as luteolin 7-*O*-rutinoside, luteolin 7-*O*-glucoside, isorhamnetin 3-*O*-glucoside, apigenin 7-*O*-glucoside, and luteolin. Neochlorogenic, chlorogenic, caffeic, 3,4-dicaffeoylquinic, 3,5-dicaffeylquinic and 1,5-dicaffeoylquinic acids were supplied from Phytolab (Vesten-bergsgreuth, Bavaria, Germany).

### 3.4. UHPLC-HRMS

The UHPLC-HRMS analyses were carried out on a Q Exactive Plus mass spectrometer (ThermoFisher Scientific, Inc., Waltham, MA, USA) equipped with a heated electrospray ionization (HESI-II) probe (ThermoScientific). The equipment was operated in negative and positive ion modes within the *m*/*z* range from 100 to 1000. The mass spectrometer parameters were as follows: spray voltage 3.5 kV (+) and 2.5 kV (−); sheath gas flow rate 38; auxiliary gas flow rate 12; spare gas flow rate 0; capillary temperature 320 °C; probe heater temperature 320 °C; S-lens RF level 50; scan mode: full MS (resolution 70,000), and MS/MS (17,500). Chromatographic separation was achieved on a reversed phase column Kromasil EternityXT C18 (1.8 µm, 2.1 × 100 mm) at 40 °C. UHPLC analyses were run with a mobile phase consisting of 0.1% formic acid in water (A) and 0.1% formic acid in acetonitrile (B). The run time was 33 min. The flow rate was 0.3 mL/min. The gradient elution program was used as follows: 0–1 min, 0–5% B; 1–20 min, 5–30% B; 20–25 min, 30–50% B; 25–30 min, 50–70% B; 30–33 min, 70–95%; 33–34 min, 95–5% B. Equilibration time was 4 min [22]. Data were processed by Xcalibur 4.2 (ThermoScientific, Waltham, MA, USA) instrument control/data handling software. MZmine 2.53 software was applied to the UHPLC–HRMS raw files of the *P. purpurea* extract for the semi-quantitative analysis. Results are expressed as the % peak area of the compound to the total peak areas of the corresponding group secondary metabolites and all metabolites.

### 3.5. The Determination of Total Polyphenols, Flavonoid Contents, and Antioxidant Activity 

The evaluation of the total polyphenols and flavonoid contents of the extract was performed as previously described via colorimetric testing using Folin–Ciocalteu and AlCl_3_, respectively [51]. To evaluate the antioxidant potential of the extract, a set of six complementary in vitro spectrophotometric tests were performed. They included free radical scavenging activity assays DPPH and ABTS, FRAP and CUPRAC tests, which evaluated the extract’s reduction capabilities, as well as metal chelating ability (MCA) and phosphomolybdenum (PBD) assays. All methods, except for MCA, were evaluated using the Trolox standard. The comparison for MCA was made in terms of equivalent EDTA equivalent per gram of extract [51].

### 3.6. In Vitro Cytotoxicity Assays

#### 3.6.1. Cell Lines and Culture Conditions

The hepatoprotective activity of the *Prenanthes extract* and the phytocompounds CA and DiCQA was assessed against diclofenac-induced cell stress in the human hepatocellular carcinoma cell line (HEP-G2), purchased from the German Collection of Microorganisms and Cell Cultures (DSMZ GmbH, Braunschweig, Germany). Cell cultures were cultivated in a growth medium RPMI 1640 supplemented with 10% fetal bovine serum (FBS) and 5% L-glutamine and incubated under standard conditions of 37 °C and 5% humidified CO_2_ atmospheres.

#### 3.6.2. MTT Cell Viability Assay

The cell viability of HEP-G2 cells following 24 h exposure to the hepatotoxic xenobiotic diclofenac was evaluated in several treatment groups subjected to a prior 24 h pretreatment with the phytoprotective substances and estimated both against untreated and unprotected control samples. The cytotoxicity was estimated with respect to diclofenac (fold-changes in the qui-effective half-inhibitory IC_50_ concentrations among treatment groups) using a validated methodology for assessing cell viability known as the Mosmann MTT assay. Exponential-phased cells were harvested and seeded (100 μL/well) in 96-well plates at the appropriate density (1.5 × 10^5^). On the following day, a pretreatment for 24 h was conducted with the *Prenanthes* extract, Chlorogenic acid, and 3,5-Dicaffeoylquinic acid in the concentration range 400–12.5 µg/mL. Both protected and unprotected treatment groups were exposed to six-fold serial dilutions of the hepatotoxin diclofenac (1600–50 µg/mL) and incubated for further 24 h. Filter sterilized MTT substrate solution (5 mg/mL in PBS) was added to each well of the culture plate. A further 2–4 h incubation allowed for the formation of purple insoluble formazan crystals. The latter were dissolved in an isopropyl alcohol solution containing 5% formic acid prior to absorbance measurement at 550 nm. The collected absorbance values were blanked against an MTT-and isopropanol solution and normalized to the mean value of untreated control (100% cell viability).

#### 3.6.3. Chou Talalay Method

The establishment and quantitative evaluation of any hepatoprotective effects by the natural products against diclofenac-induced liver cell damage were conducted using the Chou-Talalay method and its respective Compusyn^®^ software. The “dose-response” relationships were derived in advance via the standard MTT assay, according to the protocol described in the previous section. The antagonistic activity of the *Prenanthes* extract and the tested phytocompounds against the hepatotoxic agent was measured in a fixed 1:4 ratio combination, whereby preservative treatments preceded the noxious stimuli by 24 h. Promoting cellular tolerance and its degree was evaluated at each experimental data point (actual treatment concentrations), as well as at each dataset alongside the simulated “dose-response” curves. Тhe nature of the studied drug interactions was determined based on automated analysis in the Compusyn^®^ software, which generated combination (CI) and drug-reduction (DRI) indices. The CI provided a quantitative determination of a synergistic (CI < 1), additive (CI = 1), and antagonistic (CI > 1) drug behavior in fixed- or varying-ratio combinations. Similarly, a DRI (dose reduction index)-Fa plot indicated the fold-change in the equi-effective concentrations of a single drug when used in combination (DRI > 1 for synergistic interactions and 0 > DRI < 1 for antagonistic behavior). Corresponding isobolograms were also constructed and could be used as an accessory tool in evaluating drug performance.

### 3.7. Proteomic Analysis

A series of immunoassay experiments were performed to monitor proteomic changes in the expression profile of diclofenac-exposed hepatic cells subjected or not subjected to a phytoprotective pretreatment with the *Prenanthes* extract and one of its major constituents, chlorogenic acid. Changes in oxidative stress-related proteins in response to diclofenac treatment (133.3 µg/mL) were tracked and analyzed in a comparative manner to untreated control and pretreated samples (33.3 µg/mL) in membrane-based sandwich immunoassays conducted according to the manufacturer’s instructions (Proteome Profiler Human Cell Stress Array Kit, R&D Systems). The treatment doses in the fixed ratio design were selected according to the cytotoxicity data obtained from the MTT test (mean IC_50_ value of diclofenac when used alone and in combination). The proteins were visualized using a digital imaging system (Azure Biosystems C600) and densitometric analysis of the array spots was conducted using ImageJ^®^ software. The most prominent changes in spot signals were expressed graphically, relative to untreated control, and interpreted in a comparative manner to pretreated samples.

## 4. Conclusions

As a part of subtribe Hypochaeridinae, tribe Cichorieae, this is the first extensive study on the secondary metabolites of the *P. purpurea* species, further combined with a thorough evaluation of its biological activity against two caffeoylquinic acids that are relevant to its content, CA and 3,5-DiQCA, and anticipated to possess antioxidant properties. For the first time, more than 100 secondary metabolites were reported for *P. purpurea*, with polyphenolic derivatives dominating the phytochemical profile (i.e., quinic acid, chlorogenic, 3,5-dicaffeoylquinic, 5-feruloylhydroxyquinic acid, caffeoyltartaric, cichoric acids, eryodictiol-*O*-hexuronide, and luteolin *O*-hexuronide). In line with the identified compounds and the received data for the total content of polyphenols and flavonoids, the results indicate significant correlation between the presence of dihydroxycinnamic and caffeic acid derivatives (caffeoylquinic and caffeoyl tartaric acid) and the DPPH-scavenging and antioxidant capacity of the *P. purpurea* leaf extract. According to the conducted cytotoxicity studies, a 24 h incubation of hepatocyte cultures with the PE prior to diclofenac exposure exerted a positive modulating effect on the NSAID’s toxicity in a predominantly dose-dependent manner. In the highest treatment dose, the estimated DRI index for diclofenac points to a ca. three-fold increase in its equi-inhibitory concentration, implying the occurrence of moderate hepatoprotection. The protective effect of the pure polyphenolic compounds CA and 3,5-DiQCA in the combination studies was fairly more pronounced with an up to 10-fold reduction in diclofenac’s toxicity—a reasonable trend given their higher molar content in the treatment doses compared to the plant extract. According to the conducted immunoassays, cell stress response was also favorably altered in our in vitro DILI model, where CA was slightly more efficient in abrogating proapoptotic signaling, whereas the *Prenanthes* extract mainly adjusted the cellular antioxidant defense system.

## Figures and Tables

**Table 2:** The Total polyphenols, flavonoids content, and antioxidant activity of the *P. purpurea* extract.

Activity	Means ± SD
Total polyphenols mg (mg GAE/g)	35.53 ± 0.26
Total flavonoids (mg RE/g)	7.28 ± 0.11
DPPH (mg TE/g)	52.30 ± 0.36
ABTS (mg TE/g)	87.43 ± 3.58
CUPRAC (mg TE/g)	148.90 ± 3.22
FRAP (mg TE/g)	75.82 ± 1.31
Chelating (mg EDTAE/g)	40.43 ± 1.03
Phosphomolybdenum (mmol TE/g)	1.19 ± 0.03

**Table 3:** The in vitro cytotoxicity of diclofenac [µg/mL ± SD] against HEP-G2 cells when used alone or in a pretreatment regimen with PE, CA, and DiCQA.

Cell Line/ Treatment Group	Diclofenac	Diclofenac + PE	Diclofenac + CA	Diclofenac + DiCQA
HEP-G2 ^1^	90.0 ± 4.5	98.2 ± 5.3	174.1 ± 3.2	160.3 ± 2.9

^1^ human hepatocellular carcinoma cell line.

**Table 4:** DRI and CI estimates for the experimental data points of the studied combination PE + diclofenac.

Fa (Diclofenac Alone)	Fa’ (Combo)	Dose Diclofenac	Dose PE	DRI Diclofenac	CI Value
0.93	0.89	1600.0	400.0	0.35442	Infinit
0.92	0.88	800.0	200.0	0.65322	Infinit
0.92	0.85	400.0	100.0	1.05444	Infinit
0.83	0.75	200.0	50.0	1.24303	Infinit
0.55	0.5	100.0	25.0	0.99757	Infinit
0.15	0.1	50.0	12.5	0.32125	Infinit

**Table 5:** DRI and CI estimates for the experimental data points of the studied combination CA + diclofenac.

Fa (Diclofenac Alone)	Fa’ (Combo)	Dose Diclofenac	Dose CA	DRI Diclofenac	CI Value
0.93	0.66	1600.0	400.0	0.10821	Infinit
0.92	0.6	800.0	200.0	0.17467	Infinit
0.92	0.55	400.0	100.0	0.29466	Infinit
0.83	0.4	200.0	50.0	0.35609	Infinit
0.55	0.37	100.0	25.0	0.64096	Infinit
0.15	0.17	50.0	12.5	0.53409	Infinit

**Table 6:** DRI and CI estimates for the experimental data points of the studied combination 3,5-DiCQA + diclofenac.

Fa (Diclofenac Alone)	Fa’ (Combo)	Dose Diclofenac	Dose 3,5-DiCQA	DRI Diclofenac	CI Value
0.93	0.65	1600.0	400.0	0.10430	Infinit
0.92	0.62	800.0	200.0	0.18731	Infinit
0.92	0.53	400.0	100.0	0.27558	Infinit
0.83	0.52	200.0	50.0	0.53310	Infinit
0.55	0.37	100.0	25.0	0.64096	Infinit
0.15	0.01	50.0	12.5	0.04378	Infinit

## Data Availability

The data presented in this study are available in the article and the Supplementary Materials.

## Supplementary Materials

The supporting information can be downloaded at: https://www.mdpi.com/article/10.3390/ijms241814148/s1.
