# Supplementary material for: The Phytochemical Profiling, In Vitro Antioxidant, and Hepatoprotective Activity of Prenanthes purpurea L. and Caffeoylquinic Acids in Diclofenac-Induced Hepatotoxicity on HEP-G2 Cells
Source: Int J Mol Sci. 2023 Sep 15;24(18):14148. doi: 10.3390/ijms241814148 (PMC10532168; doi:10.3390/ijms241814148)
Supplement: Supplementary file 1 [file ijms-24-14148-s001.zip › ijms-2608913-supplementary.pdf]

The Phytochemical Profiling, In Vitro Antioxidant, and Hepatoprotective Activity of *Prenanthes purpurea* L. and Caffeoylquinic Acids in Diclofenac-Induced Hepatotoxicity on HEP-G2 Cells

Rositsa Mihaylova [1], Reneta Gevrenova [2], Alexandra Stefanova [1], Dimitrina Zheleva-Dimitrova [2], Vessela Balabanova [2], Gökhan Zengin [3], Rumyana Simeonova [1] and Georgi Momekov [1,*]

[1] Department of Pharmacology, Pharmacotherapy and Toxicology, Faculty of Pharmacy, Medical University of Sofia, 1000 Sofia, Bulgaria; rmihaylova@pharmfac.mu-sofia.bg (R.M.); astefanova22@gmail.com (A.S.); rsimeonova@pharmfac.mu-sofia.bg (R.S.)

[2] Department of Pharmacognosy, Faculty of Pharmacy, Medical University of Sofia, 1000 Sofia, Bulgaria; rgevrenova@pharmfac.mu-sofia.bg (R.G.); dzheleva@pharmfac.mu-sofia.bg (D.Z.-D.); vbalabanova@pharmfac.mu-sofia.bg (V.B.)

[3] Physiology and Biochemistry Research Laboratory, Department of Biology, Science Faculty, Selcuk University, Konya 42130, Turkey; gokhanzengin@selcuk.edu.tr

* Correspondence: gmomekov@pharmfac.mu-sofia.bg

Supplemental material

Table S1. Secondary metabolites in *Prenanthes purpurea* methanol-aqueous extracts

№	Identified/tentatively annotated compound	Molecular formula	Exact mass [M-H]⁻	Fragmentation pattern in (-) ESI-MS/MS	t_R (min)	Δ ppm	Level of Confidence [1]
				**Hydroxybenzoicand hydroxycinnamic acids, and phenylethanoid glycosides**			
**1.**	gallic acid[a]	$C_7H_6O_5$	169.0142	169.0130 (35.5), 125.0228 (100), 107.0122 (0.3)	1.15	-7.198	1
**2.**	gallic acid-hexoside	$C_{13}H_{15}O_{10}$	331.0678	331.0671 (100), 169.0127 (4.1), 168.0052 (34.9), 151.0019 (0.6), 149.9946 (14.48), 125.0230 (37.7), 107.0120 (0.4)	1.22	0.212	2
**3.**	hydroxybenzoic acid-*O*-hexoside	$C_{13}H_{16}O_8$	299.0778	299.0782 (0.3), 137.0229 (100), 93.0329 (68.5)	1.29	3.074	2
**4.**	protocatechuic acid-*O*-hexoside	$C_{13}H_{16}O_9$	315.0727	315.0722 (100), 153.0181 (27.5), 152.0101 (59.6), 123.0072 (2.0), 109.0286 (10.5), 108.0201 (92.5)	1.67	0.269	2
**5.**	vanillic acid-*O*-hexoside	$C_{14}H_{18}O_9$	329.0875	329.0885 (1.7),167.0337 (100), 152.0101 (20.9), 123.0436 (13.9), 108.0201 (36.4)	1.76	2.111	2
**6.**	protocatechuic acid[a]	$C_7H_6O_4$	153.0181	153.0179 (15.3), 109.0270 (100), 81.0331 (1.1)	2.04	-9.358	1
**7.**	*O*-hydroxybenzoyl-hexose	$C_{13}H_{16}O_8$	299.0778	299.0772 (100), 239.0557 (21.0), 209.0448 (6.0), 179.0339 (49.8), 137.0230 (97.6), 93.0329 (19.9)	2.06	-0.002	2
**8.**	protocatechuic acid-*O*-hexoside	$C_{13}H_{16}O_9$	315.0727	315.0723 (100), 153.0180 (61.3), 109.0279 (73.5)	2.11	0.459	2
**9.**	syringic acid-*O*-hexoside	$C_{15}H_{20}O_{10}$	359.0985	359.0987 (8.4), 197.0446 (100), 182.0210 (21.5), 166.9974 (8.8), 153.0544 (0.3), 138.0308 (29.0), 123.0072 (32.1)	2.27	0.808	2
**10.**	protocatechuic acid-hexosylpentoside	$C_{18}H_{24}O_{13}$	447.1144	447.1146 (100), 315.0726 (1.4), 153.0155 (3.1), 152.0102 (47.3), 123.0071 (1.0), 109.0288 (3.6), 108.0201 (35.1)	2.37	0.394	2
**11.**	caffeic acid *O*-hexoside	$C_{15}H_{18}O_9$	341.0871	341.0862 (3.8), 179.0338 (100), 161.0230 (0.9), 135.0437 (61.5)	2.42	-4.677	2
**12.**	hydroxybenzoic acid-*O*-hexoside	$C_{13}H_{16}O_8$	299.0778	299.0774 (13.3), 137.0230 (100), 108.0204 (0.6), 93.0330 (0.2)	2.45	0.600	2

**13.**	*O*-caffeoyl hexose	$C_{15}H_{18}O_9$	341.0871	341.0869 (28.9), 281.0668 (2.2), 251.0558 (19.5), 221.0448 (2.7), 179.0339 (44.4), 161.0231 (100), 135.0437 (24.0), 133.0281 (29.5)	2.50	-2.537	2
**14.**	gentisic acid-*O*-hexoside	$C_{13}H_{16}O_9$	315.0727	315.0725 (33.9), 153.0180 (70.0), 135.0074 (4.7), 109.0279 (100), 65.0380 (7.4)	2.58	1.316	2
**15.**	aesculetin-*O*-hexoside	$C_{15}H_{15}O_9$	339.0724	339.0722 (3.3), 177.0181 (100), 149.0235 (1.2), 133.0280 (8.2), 105.0330 (3.7), 89.0381 (2.2),	2.73	0.515	2
**16.**	4-hydroxybenzoic acid	$C_7H_6O_3$	137.0230	137.0229 (100), 119.0124 (2.5), 108.0200 (10.3), 93.0329 (2.6), 65.0380 (1.7)	2.85	-10.709	2
**17.**	hydroxybenzoic acid-*O*-hexoside	$C_{13}H_{16}O_8$	299.0778	299.0780 (1.7), 137.0229 (100), 93.0329 (47.3)	3.01	2.539	2
**18.**	caffeic acid *O*-hexoside	$C_{15}H_{18}O_9$	341.0871	341.0877 (27.0), 179.0338 (100), 161.0232 (1.7), 135.0436 (72.0)	3.08	-0.367	2
**19.**	*p*-coumaric acid[a]	$C_9H_8O_3$	163.0389	163.0388 (10.4), 135.0431 (1.1), 119.0487 (100)	3.10	-7.835	1
**20.**	umbelliferone	$C_9H_6O_3$	161.0244	161.0231 (79.0), 133.0280 (100), 115.0174 (1.8), 105.0330 (1.8), 89.0380 (1.1), 77.0381 (1.0)	3.16	-8.057	2
**21.**	quinic acid	$C_7H_{12}O_6$	191.0549	191.0550 (100), 173.0444 (1.8), 155.0338 (0.2), 127.0386 (3.5), 111.0435 (1.7), 93.0330 (6.3), 85.0278 (19.4)	3.19	-5.921	2
**22.**	vanillic acid *O*-deoxyhexoside	$C_{14}H_{18}O_8$	313.0929	313.0930 (11.0), 167.0337 (100), 152.0102 (16.8), 123.0436 (24.1), 108.0201 (29.1),	3.26	0.509	2
**23.**	coumaric acid-*O*-hexoside	$C_{15}H_{18}O_8$	325.0930	325.0929 (6.3), 163.0385 (100), 145.0281 (31.2), 119.0487 (96.0), 135.0434 (0.4)	3.33	-1.386	2
**24.**	syringic acid-(caffeoyl)-pentoside	$C_{22}H_{20}O_{13}$	491.0831	491.0831 (8.5), 329.0514 (62.4), 197.0446 (13.9), 179.0338 (21.8), 161.0234 (4.5), 153.0544 (27.7)	3.34	-0.028	2
**25.**	*m*-coumaric acid[a]	$C_9H_8O_3$	163.0389	163.0387 (1.8), 135.0436 (0.9), 119.0487 (100)	3.35	-8.203	1
**26.**	aesculetin	$C_9H_6O_4$	177.0193	177.0181 (100), 149.0232 (3.1), 133.0280 (20.6), 105.0330 (10.7), 89.0380 (7.1)	3.46	-6.790	2
**27.**	caffeic acid[a]	$C_9H_8O_4$	179.0339	179.0339 (20.5), 135.0437 (100), 117.0328 (0.4), 107.0486 (1.5)	3.55	-5.810	1
**28.**	syringic acid *O*-deoxyhexoside	$C_{15}H_{20}O_9$	343.1035	343.1036 (22.6), 197.0447 (100), 182.0210 (18.7), 166.9974 (6.3), 153.0594 (3.5), 138.0308 (19.6), 123.0072 (23.7)	3.65	0.363	2
**29.**	caffeoylmalic acid	$C_{13}H_{12}O_8$	295.0459	259.0490 (1.1), 179.0339 (12.1), 161.0228 (0.2), 135.0437 (8.80, 133.0124 (100), 115.0021 (26.2), 89.0228 (1.8), 72.9915 (2.5), 71.0122 (10.1)	4.18	0.676	2
**30.**	coumaric acid-*O*-hexoside	$C_{15}H_{18}O_8$	325.0930	325.0930 (1.4), 163.0387 (98.4), 135.0435 (0.2), 119.0487 (100)	4.46	0.490	2

**31.**	*o*-coumaric acid[a]	$C_9H_8O_3$	163.0389	163.0386 (7.7), 119.0486 (100)	4.58	-8.326	1
**32.**	caffeoylcitramalic acid	$C_{14}H_{14}O_8$	309.0616	309.0622 (1.6), 179.0337 (2.6), 161.0228 (2.0), 147.0285 (100), 129.0178 (40.7), 101.0228 (6.8), 85.0279 (10.2)	4.76	1.745	2
**33.**	vanillic acid[a]	$C_8H_8O_4$	167.0338	167.0337 (100), 152.0102 (26.2), 124.0151 (10.0), 108.0203 (0.9)	4.79	-7.376	1
**34.**	gallic acid-(caffeoyl)-hexoside	$C_{22}H_{22}O_{13}$	493.0988	493.0991 (100), 331.0672 (62.7), 179.0339 (2.4), 169.0127 (3.1), 168.0052 (28.8), 149.9945 (19.5), 135.0437 (4.50, 133.0281 (6.4), 125.0229 (24.2)	5.18	0.661	2
**35.**	protocatechuic acid – (caffeoyl)-hexoside 1	$C_{22}H_{22}O_{12}$	477.1038	477.1038 (100), 315.0723 (44.5), 179.0339 (3.4), 161.0231 (33.8), 153.0181 (10.4), 152.0101 (30.7), 135.0437 (6.2), 133.0280 (17.1), 123.0073 (1.1), 109.0284 (5.5), 108.0201 (32.6)	5.67	-0.124	2
**36.**	protocatechuic acid – (caffeoyl)-hexoside 2	$C_{22}H_{22}O_{12}$	477.1038	477.1034 (100), 315.0725 (71.8), 179.0336 (2.8), 161.0231 (10.1), 153.0180 (52.4), 135.0438 (6.6), 133.0280 (6.3), 123.0071 (1.9), 109.0279 (56.9)	5.81	-0.900	2
**37.**	salicylic acid[a]	$C_7H_6O_3$	137.0230	137.0229 (10.0), 108.0206 (0.8), 93.0329 (100), 65.0380 (1.3)	6.27	-10.928	1
**38.**	protocatechuic acid – (caffeoyl)-hexoside	$C_{22}H_{22}O_{12}$	477.1038	477.1039 (64.71), 315.0719 (5.6), 179.0341 (2.3), 161.0231 (23.8), 153.0179 (100), 135.0436 (5.3), 133.0280 (11.5), 109.0279 (40.0)	6.54	0.002	2
**39.**	caffeic acid-(hydroxybensoyl)-hexoside	$C_{22}H_{22}O_{11}$	461.1089	461.1095 (48.2), 323.0771 (24.7), 179.0335 (5.3), 161.0231 (21.2), 137.0229 (100), 135.0437 (6.2), 133.0280 (10.0), 93.0330 (54.8)	6.60	1.226	2
				**Mono- and diacylquinic acids, and derivatives**			
**40.**	neochlorogenic (3-caffeoylquinic) acid[a]	$C_{16}H_{18}O_9$	353.0867	353.0880 (40.8), 191.0551 (100), 179.0339 (61.6), 173.0440 (3.7), 161.0231 (3.9), 135.0437 (50.5), 127.0385 (2.0)	2.36	0.410	1
**41.**	4-feruloylquinic acid-hexoside	$C_{23}H_{30}O_{14}$	529.1563	529.1567 (100), 367.1034 (1.1), 337.0952 (0.2), 193.0497 (8.7), 191.0551 (9.9), 178.0262 (3.7), 173.0443 (41.5), 149.0594 (2.3), 134.0359 (12.9), 111.0436 (10.5), 93.0329 (79.6),	2.64	0.872	2
**42.**	5-caffeoyl-2-hydroxyquinic acid	$C_{16}H_{18}O_{10}$	369.0827	369.0826 (2.7), 207.0502 (100), 179.0334 (1.9), 189.0385 (0.3), 135.0439 (2.0), 109.0279 (2.6), 101.0227 (1.5), 85.0278 (5.0)	2.82	-0.379	2
**43.**	chlorogenic (5-caffeoylquinic) acid[a]	$C_{16}H_{18}O_9$	353.0874	353.0873 (5.0), 191.0550 (100), 179.0334 (1.0), 173.0446 (0.4), 161.0229 (1.7), 135.0433 (1.1), 127.0384 (2.1)	3.18	-1.403	1

**44.**	4-caffeoylquinic acid	$C_{16}H_{18}O_9$	353.0878	353.0880 (28.0), 191.0551 (100), 179.0339 (54.6), 173.0444 (82.4), 135.0437 (43.7), 127.0384 (1.6), 111.0435 (3.2), 93.0330 (20.8), 85.0279 (12.2)	3.37	0.495	2
**45.**	5-*p*-coumaroyl-2-hydroxyquinic acid 2	$C_{16}H_{18}O_9$	353.0878	353.0876 (12.8), 207.0502 (100), 189.0395 (5.8), 163.0387 (5.3), 127.0384 (4.3), 109.0280 (8.0), 101.0229 (4.6), 85.0279 (9.1)	3.59	-0.581	2
**46.**	5-feruloylquinic acid-hexoside	$C_{23}H_{30}O_{14}$	529.1563	529.1567 (100), 367.1034 (5.4), 193.0497 (14.9), 191.0551 (75.2), 178.0260 (4.7), 173.0444 (19.8), 149.0597 (2.4), 134.0359 (11.6), 127.0384 (1.5), 111.0436 (6.5), 93.0330 (41.9)	3.61	0.872	2
**47.**	3-feruloylquinic acid	$C_{17}H_{20}O_9$	367.1034	367.1075 (10.9), 191.0551 (100), 173.0444 (47.9), 93.0330 (40.3), 85.0279 (11.1)	3.71	4.954	2
**48.**	5-feruloylquinic acid-pentoside	$C_{22}H_{28}O_{13}$	499.1457	499.1464 (100), 367.1031 (12.0), 193.0494 (8.0), 191.0551 (71.2), 178.0256 (4.3), 173.0444 (28.4), 149.0595 (1.9), 134.0359 (6.2), 111.0436 (8.7), 93.0330 (55.7), 85.02798 (3.0)	3.73	1.414	2
**49.**	5-caffeoyl-2-hydroxyquinic acid 2	$C_{16}H_{18}O_{10}$	369.0827	369.0819 (3.8), 207.0501 (100), 191.0551 (5.0), 179.0348 (0.8), 161.0233 (0.2), 135.0179 (0.7), 109.0279 (2.9), 85.0278 (5.7)	2.82	-0.379	2
**50.**	5-feruloyl-hydroxyquinic acid-hexosylpentoside	$C_{28}H_{38}O_{19}$	677.1929	677.1942 (100), 383.0986 (1.4), 207.0502 (92.1), 193.0496 (0.5), 189.0394 (20.3), 178.0262 (3.8), 171.0287 (0.4), 134.0357 (8.3), 127.0386 (10.5), 109.0279 (23.6), 101.0228 (9.8), 85.0278 (17.7)	3.19	1.119	2
**51.**	5-caffeoylquinic acid isomer	$C_{16}H_{18}O_9$	353.0874	353.0879 (5.4), 191.0551 (100), 179.0335 (0.9), 161.0232 (2.6), 127.0384(1.7), 93.0329 (2.8), 85.0278 (8.4)	3.90	0.155	2
**52.**	5-*p*-coumaroylquinic acid	$C_{16}H_{18}O_8$	337.0928	337.0930 (8.7), 191.0551 (100), 173.0443 (6.8), 163.0388 (6.2), 145.0278 (1.3), 127.0385 (0.9), 119.0489 (4.1), 111.0436 (2.1), 93.0329 (15.4), 85.0278 (4.4)	3.98	0.473	2
**53.**	3-caffeoyl-5-hydroxy-dihydrocaffeoylquinic acid	$C_{25}H_{26}O_{13}$	533.1288	533.1304 (76.0), 371.0989 (11.3), 353.0883 (19.2), 335.0764 (3.7), 191.0551 (100), 179.0338 (48.7), 173.0444 (15.5), 161.0230 (7.5), 135.0436 (61.0), 127.0387 (2.8), 111.0434 (2.9), 93.0328 (7.4), 85.0278 (6.4)	4.03	0.574	2
**54.**	5-feruloyl-2-hydroxyquinic acid	$C_{17}H_{20}O_{10}$	383.0986	383.0986 (23.1), 207.0502 (100), 189.0395 (8.7), 193.0499 (4.8), 149.0592 (0.3), 134.0359 (9.5), 143.0336 (0.7), 127.0386 (5.4), 109.0279 (13.5), 101.0228 (5.7), 85.0279 (10.1)	4.11	0.600	2

**55.**	5-feruloyl-hydroxyquinic acid-hexosylpentoside	$C_{28}H_{38}O_{19}$	677.1929	677.1943 (100), 383.0986 (2.1), 207.0503 (88.8), 193.0496 (35.9), 189.0394 (16.2), 178.0262 (7.5), 149.0594 (4.1), 134.0357 (21.7), 127.0386 (8.9), 109.0279 (20.6), 101.0227 (10.6), 85.0278 (16.8)	4.14	3.11	2
**56.**	5-feruloylquinic acid	$C_{17}H_{20}O_{9}$	367.1034	367.1034 (17.4), 193.0496 (5.4), 191.0551 (100), 173.0444 (8.9), 134.0360 (9.9), 111.0437 (3.6), 93.0329 (25.5)	4.41	-0.260	2
**57.**	1-caffeoyl-3-hydroxy-dihydrocaffeoylquinic acid	$C_{25}H_{26}O_{13}$	533.1288	533.1304 (19.7), 371.0986 (61.2), 353.0885 (3.4), 335.0781 (2.5), 197.0451 (2.3), 191.0550 (15.8), 179.0338 (12.0), 173.0443 (17.0), 161.0228 (2.5), 135.0436 (100), 93.0329 (6.1), 85.0278 (3.2)	4.45	0.540	2
**58.**	5-p-coumaroyl-2-hydroxyquinic acid 2	$C_{16}H_{18}O_{9}$	353.0878	353.0876 (4.5), 207.0501 (100), 189.0396 (0.6), 163.0389 (1.3), 127.0386 (1.9), 109.0280 (2.7), 101.0227 (2.5), 85.0278 (6.0)	4.50	-0.723	2
**59.**	5-feruloylquinic acid-pentoside	$C_{22}H_{28}O_{13}$	499.1457	499.1460 (100), 367.1036 (4.9), 193.0497 (11.8), 191.0551 (99.4), 178.0260 (4.1), 173.0444 (15.7), 149.0596 (1.9), 134.0359 (9.6), 127.0386 (1.8), 111.0436 (4.4), 93.0329 (32.7), 85.02778 (7.0)	4.51	0.573	2
**60.**	5-*p*-coumaroylquinic acid isomer	$C_{16}H_{18}O_{8}$	337.0928	337.0928 (5.8), 191.0550 (100), 173.0448 (1.8), 163.0387 (1.4), 127.0386 (1.6), 119.0485 (0.8), 111.0434 (1.0), 93.0330 (3.9), 85.0278 (5.7)	4.63	-0.180	2
**61.**	5-feruloyl-2-hydroxyquinic acid	$C_{17}H_{20}O_{10}$	383.0985	383.0985 (6.9), 207.0501 (100), 193.0499 (1.3), 127.0386 (2.3), 109.0279 (8.2), 101.0229 (2.8), 85.0279 (6.0)	4.76	0.600	2
**62.**	1, 3-dicaffeoylquinic acid-hexoside	$C_{31}H_{34}O_{17}$	677.1723	677.1732 (75.3), 515.1407 (67.0)), 497.1298 (2.0), 353.0878 (19.3), 341.0881 (23.8), 335.0777 (9.1), 323.0771 (13.7), 227.0744 (0.4), 191.0551 (76.4), 179.0339 (94.7), 173.0446 (8.9), 161.0231 (23.6), 135.0437 (100), 111.0435 (1.9), 93.0330 (6.7), 85.0279 (7.2)	5.16	1.325	2
**63.**	3,4-dicaffeoylquinic acid[a]	$C_{25}H_{24}O_{12}$	515.1190	515.1196 (100), 353.0879 (13.6), 335.0767 (6.8), 203.0338 (0.4), 191.0551 (28.8), 179.0338 (52.0), 173.0443 (61.7), 161.0231 (16.9), 135.0436 (55.7), 111.0434 (5.0), 93.0328 (17.0), 85.0276 (4.3)	5.70	0.254	1
**64.**	3,5-dicaffeoylquinic acid[a]	$C_{25}H_{24}O_{12}$	515.1189	515.1188 (20.2), 353.0876 (100), 335.0761 (0.5), 191.0550 (79.6), 179.0338 (39.9), 161.0232 (3.8), 135.0436 (49.6), 127.0385 (3.0), 111.0434 (1.7), 93.0329 (3.8), 85.0277 (7.3)	5.85	-1.416	1
**65.**	1,5-dicaffeoylquinic acid[a]	$C_{25}H_{24}O_{12}$	515.1190	515.1198 (30.2), 353.0879 (100), 335.0778 (2.6), 191.0551 (91.6), 179.0339 (49.3), 173.0443 (8.6),	6.02	0.720	1

				161.0231 (5.7), 135.0436 (57.7), 127.0386 (2.3), 111.0433 (1.6), 93.0329 (5.0), 85.0278 (8.8)			
**66.**	4,5-dicaffeoylquinic acid	$C_{25}H_{24}O_{12}$	515.1190	515.1196 (100), 353.0878 (70.0), 335.0774 (2.2), 203.0341 (3.2), 191.0550 (37.1), 179.0339 (69.1), 173.0443 (97.4), 135.0437 (62.1), 127.0388 (2.4), 111.0435 (4.1), 93.0329 (25.2), 85.0278 (4.3)	6.23	0.196	2
**67.**	3-*p*-coumaroyl-5-caffeoylquinic acid	$C_{25}H_{24}O_{11}$	499.1251	499.1245 (17.6), 353.0894 (0.3), 337.0930 (74.8), 335.0787 (2.4), 191.0551 (8.8), 173.0444 (7.3), 163.0387 (100), 161.0233 (3.2), 135.0436 (2.2), 127.0386 (1.2), 119.0487 (37.7), 111.0440 (1.3), 93.0330 (2.8), 85.0279 (0.9)	6.52	-0.210	2
**68.**	3-caffeoyl-5-*p*-coumaroylquinic acid	$C_{25}H_{24}O_{11}$	499.1251	499.1245 (25.6), 353.0878 (64.4), 337.0930 (23.7), 191.0550 (100), 179.0338 (37.1), 173.0444 (8.9), 163.0388 (8.7), 161.0231 (6.3), 145.0283 (2.0), 135.0437 (38.3), 127.0385 (1.8), 119.0487 (5.4), 111.0434 (2.0), 93.0329 (11.9), 85.0278 (6.0)	6.58	-0.150	2
**69.**	1-*p*-coumaroyl-5-caffeoylquinic acid	$C_{25}H_{24}O_{11}$	499.1251	499.1242 (26.2), 353.0878 (44.5), 337.0930 (43.5), 335.0768 (1.1), 191.0550 (100), 179.0338 (32.8), 173.0443 (11.6), 163.0387 (38.3), 161.0232 (0.4), 145.0283 (2.0), 135.0436 (35.4), 119.0486 (16.6), 111.0435 (2.5), 93.0329 (7.9), 85.0278 (8.7)	6.79	-0.751	2
**70.**	4-caffeoyl-5-*p*-coumaroylquinic acid	$C_{25}H_{24}O_{11}$	499.1252	499.1245 (87.1), 353.0877 (56.8), 337.0933 (15.5), 191.0551 (51.3), 179.0338 (57.9), 173.0444 (100), 163.0385 (2.7), 161.0227 (6.4), 145.0280 (1.0), 135.0437 (56.0), 127.0388 (0.9), 119.0488 (2.5), 111.0435 (4.3), 93.0330 (27.5), 85.0277 (4.7)	6.98	-0.210	2
				**Acyltartaric acids and derivatives**			
**71.**	caffeoyltartaric acid 1	$C_{13}H_{12}O_9$	311.0409	311.0413 (0.4), 179.0340 (13.8), 149.0078 (100), 135.0437 (11.9), 103.0021 (2.4), 87.0071 (13.5), 59.0122 (2.6)	2.16	1.495	2
**72.**	caffeoyltartaric acid 2	$C_{13}H_{12}O_9$	311.0409	179.0339 (63.3), 149.0078 (100), 135.0437 (45.0), 112.9865 (1.8), 103.0020 (2.2), 87.0071 (12.9), 59.0122 (2.1)	2.36	1.495	2
**73.**	*p*-coumaroyltartaric caid	$C_{13}H_{12}O_8$	295.0459	295.0461 (0.8), 163.0387 (100), 149.0074 (1.5), 135.0436 (0.6), 119.0487 (27.2), 112.9864 (8.3), 87.0071 (5.7), 59.0124 (2.7)	3.10	0.676	2
**74.**	dicaffeoyltartaric acid-hexoside	$C_{28}H_{28}O_{17}$	635.1254	635.1239 (15.9), 473.0938 (71.0), 455.0848 (13.0), 341.0877 (53.0), 311.0364 (1.5), 293.0302 (39.5), 219.0293 (17.5), 179.0339 (100), 161.0234 (11.4),	4.32	-2.318	2

				149.0078 (14.9), 135.0437 (96.4), 112.9865 (21.7), 103.0022 (0.8), 87.0070 (9.2), 59.0122 (2.3)			
**75.**	caffeoyl-dihydrocaffeoyltartaric acid	$C_{22}H_{20}O_{12}$	475.0882	475.0893 (10.1), 313.0565 (100), 293.0305 (19.9), 277.0355 (1.9), 219.0293 (11.3), 181.0494 (95.6), 179.0339 (18.1), 161.0232 (7.4), 137.0593 (17.0), 135.0430 (32.2), 112.9864 (66.9), 87.0071 (8.4), 59.0123 (15.4)	4.83	2.359	2
**76.**	cichoric acid 1	$C_{22}H_{18}O_{12}$	473.0725	473.0721 (5.4), 311.0409 (84.2), 293.0304 (21.6), 275.0183 (0.5), 231.0296 (0.6), 219.0293 (7.1), 179.0339 (78.2), 161.0226 (4.1), 149.0075 (100), 135.0437 (72.0), 103.0019 (2.6), 87.0071 (16.0), 59.0122 (2.3)	4.91	-0.949	2
**77.**	cichoric acid 2	$C_{22}H_{18}O_{12}$	473.0725	473.0723 (4.1), 311.0409 (75.9), 293.0304 (15.6), 219.0292 (5.7), 179.0338 (46.9), 161.0230 (4.0), 149.0077 (100), 135.0436 (49.0), 103.0019 (2.5), 87.0071 (17.2), 59.0122 (2.9)	5.22	-0.590	2
**78.**	*p*-coumaroyl-caffeoltartaric acid 1	$C_{22}H_{18}O_{11}$	457.0776	457.0786 (2.3), 295.0457 (82.7), 277.0352 (30.4), 231.0295 (2.4), 219.0291 (21.4), 179.0338 (36.9), 163.0387 (100), 149.0077 (9.2), 135.0436 (50.3), 119.0487 (40.5), 112.9864 (33.1), 103.0022 (3.2), 87.0071 (13.5), 59.01221 (2.4)	5.72	2.069	2
**79.**	tricaffeoyltartaric acid	$C_{31}H_{24}O_{15}$	635.1042	635.1051 (23.3), 473.0727 (100), 341.0666 (75.4), 323.0558 (3.5), 297.0768 (28.5), 293.0304 (42.5), 219.0293 (15.6), 179.0338 (15.9), 161.0232 (14.9), 149.0078 (1.1), 145.0281 (19.4), 135.0437 (29.0), 112.9865 (98.9), 87.0071 (7.8), 59.0123 (0.8)	5.99	1.349	2
**80.**	caffeoyl-feruloyltartaric acid 1	$C_{23}H_{20}O_{12}$	487.0882	487.0869 (5.5), 325.0567 (100), 307.0459 (35.9), 293.0304 (61.5), 233.0450 (18.6), 219.0290 (30.0), 193.0496 (81.8), 179.0338 (65.1), 161.0231 (19.0), 135.0437 (68.0), 134.0358 (56.3), 112.9864 (48.1), 103.0019 (3.1), 87.0071 (14.7), 59.0122 (7.9)	6.05	-2.647	2
**81.**	*p*-coumaroyl-caffeoltartaric acid 2	$C_{22}H_{18}O_{11}$	457.0776	457.0763 (3.5), 295.0457 (100), 277.0352 (19.9), 231.0292 (1.2), 219.0292 (12.7), 179.0340 (30.2), 163.0388 (95.7), 149.0078 (37.4), 145.0280 (5.4), 135.0436 (40.6), 119.0487 (41.8), 112.9865 (30.8), 103.0020 (4.0), 87.0071 (15.1), 59.0122 (4.9)	6.10	-3.007	2
**82.**	*p*-coumaroyl-caffeoltartaric acid 3	$C_{22}H_{18}O_{11}$	457.0776	457.0699 (5.5), 295.0458 (100), 277.0352 (15.4), 231.0292 (2.4), 219.0294 (13.5), 179.0338 (33.9), 163.0388 (81.3), 149.0078 (84.9), 135.0437 (38.5),	6.65	-17.034	2

				119.0487 (43.7), 112.9864 (35.0), 103.0018 (5.6), 87.0071 (21.7), 59.0122 (9.1)			
**83.**	caffeoyl-feruloyltartaric acid 2	$C_{23}H_{20}O_{12}$	487.0882	487.0887 (46.6), 325.0565 (91.3), 293.0299 (3.3), 219.0290 (30.0), 193.0496 (81.8), 179.0338 (65.1), 163.0235 (100), 161.0231 (56.8), 145.0126 (18.9), 135.0437 (23.0), 134.0360 (1.3), 112.9865 (3.7), 103.0020 (19.1), 87.0070 (3.5), 59.0122 (17.7)	7.87	0.926	2
				**Flavonoids**			
**84.**	luteolin *O*-hexosyl-(1→6)-hexoside (gentiobioside)	$C_{27}H_{30}O_{16}$	609.1464	609.1466 (67.7), 285.0403 (100), 256.0379 (1.0), 241.0501 (0.5), 217.0501 (1.4), 199.0385 (2.1), 175.0388 (1.6), 151.0021 (4.0), 133.0280 (5.6), 121.0280 (0.5), 107.0123 (2.0)	4.71	0.414	2
**85.**	luteolin *O*-hexosyl-*O*-hexuronide	$C_{27}H_{28}O_{17}$	623.1254	623.1259 (66.0), 503.0822 (1.1), 461.0730 (7.7), 327.0500 (1.9), 285.0402 (100), 267.0302 (0.2), 256.0375 (0.9), 241.0504 (1.1), 229.0502 (0.2), 217.0501 (1.8), 199.0391 (2.3), 175.0386 (2.9), 151.0022 (5.7), 133.0280 (6.4), 107.0123 (1.6)	4.84	0.766	2
**86.**	luteolin *O*-pentosyl-(1→2)-hexoside	$C_{26}H_{28}O_{15}$	579.1355	579.1359 (77.4), 285.0403 (100), 256.0371 (0.5), 241.0494 (1.0), 217.0494 (1.5), 199.031 (2.6), 175.0390 (2.3), 151.0023 (4.5), 133.0280 (6.2), 107.0122 (2.1)	5.15	0.547	2
**87.**	isorhamnetin *O*-pentosyl-(1→2)-hexoside	$C_{27}H_{30}O_{16}$	609.1464	609.1464 (100), 315.0501 (22.1), 314.0433 (78.7), 299.0197 (18.3), 285.0403 (4.6), 271.0246 (20.8), 255.0294 (5.7), 243.0294 (18.4), 227.0344 (4.6), 215.0340 (2.8), 199.0392 (5.2), 178.9981 (1.5), 165.0183 (0.3), 151.0024 (3.7), 107.0122 (0.8)	5.16	0.414	2
**88.**	luteolin 7-*O*-rutinoside[a]	$C_{27}H_{30}O_{15}$	593.1512	593.1514 (88.8), 285.0402 (100), 256.0375 (1.2), 241.0504 (0.5), 227.0354 (0.3), 217.0497 (1.0), 199.0390 (2.0), 175.0387 (2.1), 151.0023 (4.4), 133.0280 (5.3), 121.0280 (0.4), 107.0123 (2.2)	5.22	0.399	1
**89.**	apigenin *O*-hexosyl-(1→6)-hexoside (gentiobioside)	$C_{27}H_{30}O_{15}$	593.1512	593.1515 (34.2), 269.0453 (100), 241.0490 (0.1), 225.0546 (1.3), 213.0549 (0.1), 197.0593 (0.8), 151.0019 (0.9), 117.0331 (3.6), 107.0124 (1.7)	5.30	0.500	2
**90.**	eryodictiol O-hexuronide	$C_{21}H_{20}O_{12}$	463.0882	463.0883 (100), 287.0560 (45.6), 175.0236 (14.6), 151.0023 (71.3), 135.0437 (58.6), 125.0230 (4.4), 107.0123 (16.4)	5.34	0.218	2
**91.**	luteolin-*O*-hexuronide	$C_{21}H_{18}O_{12}$	461.0736	461.0727 (63.7), 285.0403 (100), 257.0450 (0.4), 243.0295 (0.5), 229.0507 (0.3), 217.0497 (1.0), 199.0391 (2.2), 175.0384 (4.7), 151.0024 (4.7), 133.0281 (8.8), 121.0278 (1.0), 107.0122 (2.8)	5.37	0.306	2

**92.**	luteolin 7-*O*-glucoside[a]	$C_{21}H_{19}O_{11}$	447.0934	447.0933 (100), 285.0402 (94.8), 284.0326 (35.8), 267.0283 (0.2), 256.0373 (3.9), 241.0499 (0.5), 227.0346 (2.2), 211.0390 (1.7), 199.0386 (1.5), 175.0391 (1.8), 151.0021 (6.2), 133.0280 (4.3), 121.0272 (0.3), 107.0122 (3.0)	5.41	0.079	1
**93.**	quercetin *O*-acetylhexoside	$C_{23}H_{22}O_{13}$	505.0988	505.0992 (100), 463.0915 (1.3), 301.0346 (36.1), 300.0274 (86.1), 271.0247 (41.5), 255.0295(17.4), 243.0294 (10.7), 227.0341 (3.0), 211.0388 (1.1), 199.0384 (1.2), 178.9978 (2.9), 163.0024 (2.0), 151.0023 (6.8), 121.0280 (1.2), 107.0123 (2.0)	5.62	0.824	2
**94.**	isorhamnetin 3-*O*-glucoside[a]	$C_{22}H_{21}O_{12}$	477.1044	477.1048 (100), 315.0487 (10.3), 314.0433 (54.4), 299.0198 (3.7), 285.0405 (8.0), 271.0246 (19.6), 257.0452 (4.3), 243.0294 (20.8), 227.0341 (3.3), 215.0351 (2.8), 199.0389 (2.7), 151.0024 (3.1), 107.0115 (0.5)	6.02	2.056	1
**95.**	apigenin 7-*O*-glucoside[a]	$C_{21}H_{19}O_{10}$	431.0980	431.0981 (100), 269.0449 (24.2), 268.0375 (55.6), 211.0389 (1.8), 171.0444 (1.4), 151.0020 (3.9), 117.0331 (1.7), 107.0121 (1.9)	6.08	-0.881	1
**96.**	apigenin-*O*-hexuronide	$C_{21}H_{18}O_{11}$	445.0774	445.0779 (24.7), 269.0455 (100), 225.0554 (0.5), 201.0548 (0.4), 175.0235 (15.7), 151.0022 (2.1), 117.0329 (6.7), 107.0122 (2.9)	6.12	-0.617	2
**97.**	naringenin *O*-hexuronide	$C_{21}H_{20}O_{11}$	447.0933	477.0925 (65.9), 271.0613 (100), 175.0236 (49.8), 151.0022 (44.2), 119.0487 (38.2), 107.0122 (18.3)	6.16	-1.755	2
**98.**	luteolin O-malonylhexoside	$C_{24}H_{22}O_{14}$	533.0939	533.0939 (0.4), 489.1041 (100), 447.0941 (1.8), 327.0499 (0.9), 285.0403 (60.9), 267.0287 (0.5), 256.0373 (0.4), 227.0345 (0.8), 199.0391 (0.7), 175.0390 (0.5), 151.0023 (3.8), 133.0278 (3.0), 107.0122 (2.4)	6.18	0.472	2
**99.**	luteolin *O*-acetylhexoside	$C_{23}H_{22}O_{12}$	489.1038	489.1038 (100), 447.0925 (0.9), 429.0792 (0.2), 285.0401 (73.6), 267.0293 (0.3), 256.0370 (3.8), 239.0345 (0.6), 227.0343 (2.1), 211.0391 (1.0), 199.0393 (1.8), 175.0385 (1.2), 151.0023 (4.1), 133.0280 (3.7), 121.0276 (0.6), 107.0122 (3.3)	6.19	0.101	2
**100.**	kaempferol *O*-acetylhexoside	$C_{23}H_{22}O_{12}$	489.1038	489.1040 (100), 285.0401 (69.2), 284.0326 (61.3), 255.0296 (35.4), 227.0344 (21.9), 211.0394 (2.0), 163.0022 (1.4), 151.0022 (2.7), 135.0071 (1.3), 107.0121 (2.0)	6.28	0.370	2
**101.**	luteolin *O*-hexoside	$C_{21}H_{19}O_{11}$	447.0934	447.0933 (60.9), 285.0401 (100), 256.0368 (2.4), 241.0498 (1.0), 225.1122 (2.3), 217.0501 (1.7),	6.41	0.079	2

				199.0381 (2.4), 175.0388 (3.6), 151.0022 (6.6), 133.0280 (11.4), 107.0123 (2.9)			
**102.**	naringenin O-acetylhexoside	$C_{23}H_{24}O_{11}$	475.1246	475.1263 (16.9), 271.0611 (100), 177.0180 (2.9), 165.0179 (1.0), 151.0023 (19.1), 119.0487 (21.1), 107.0122 (8.4)	6.89	3.505	2
**103.**	luteolin[a]	$C_{15}H_9O_7$	285.0406	285.0403 (100), 229.0494 (0.3), 217.0502 (1.2), 175.0392 (2.5), 151.0025 (4.7), 133.0280 (21.5), 121.0279 (1.0), 107.0121 (3.5)	7.58	0.346	1

[a] identified by comparison with an authentic standard; level of confidence: 1—compound identified by comparison to the reference standard; 2—putatively annotated compound; and 3—putatively characterized compound classes.

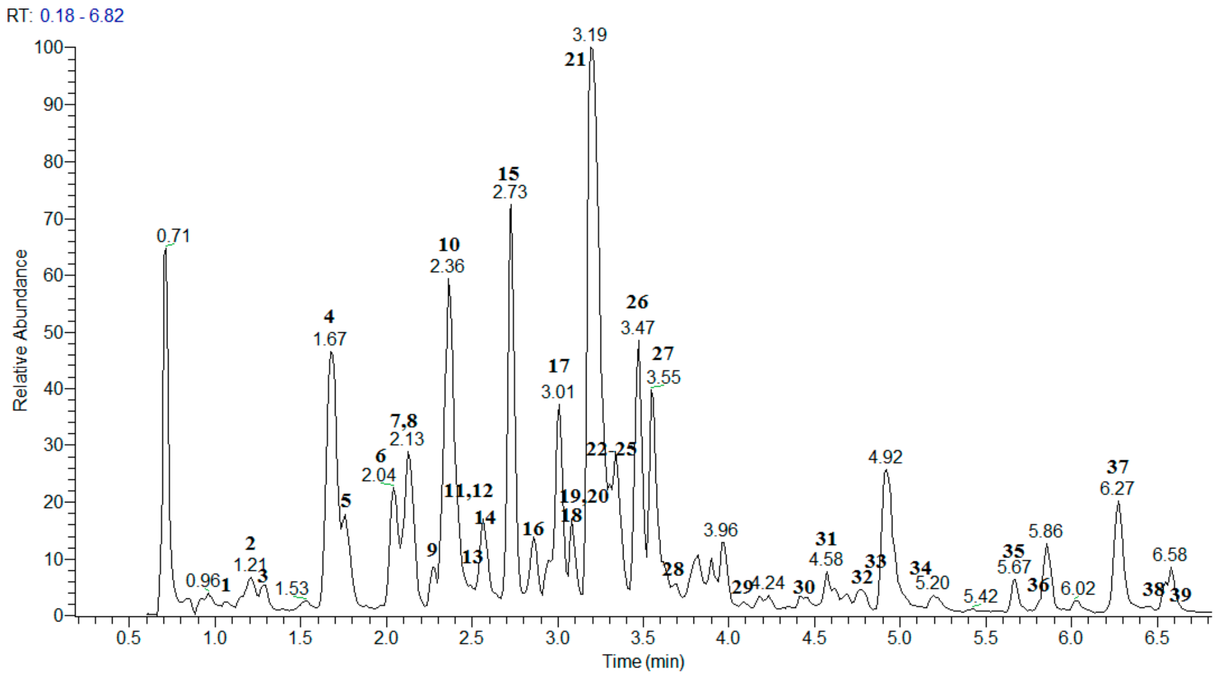

Figure S1. Extracted ion chromatograms of hydroxybenzoic and hydroxycinnamic acids and their derivatives (for numbers and fragmentation patterns, see Table S1).

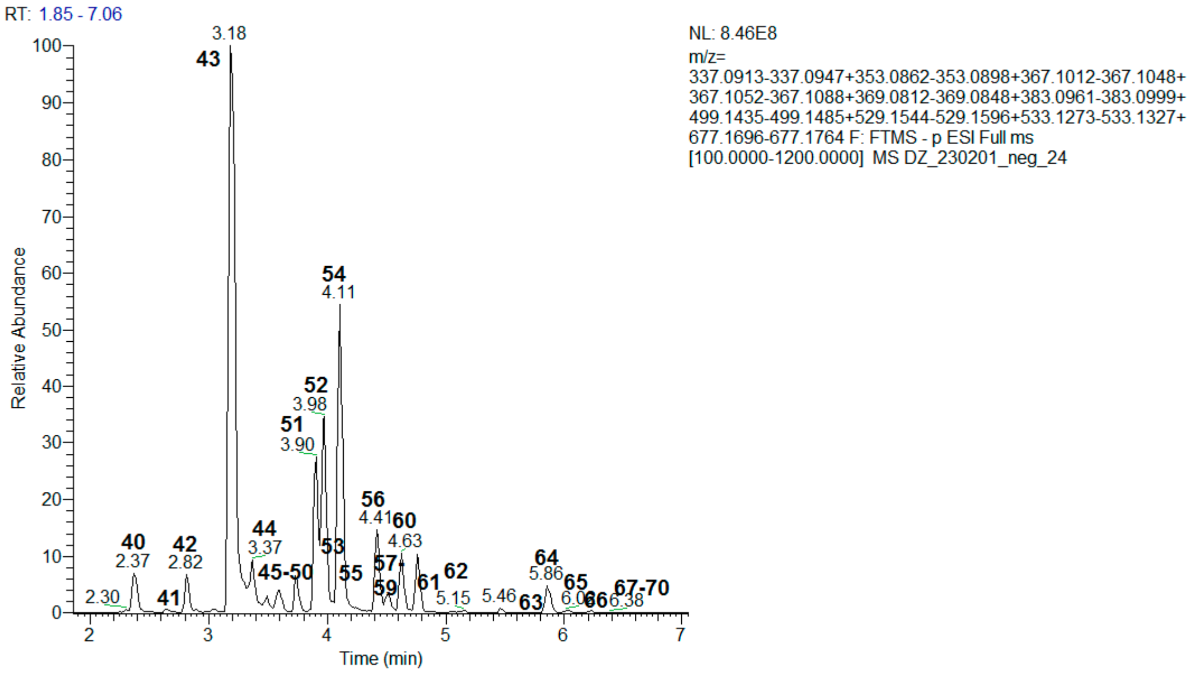

Figure S2. Extracted ion chromatograms of acylquinic acids (for numbers and fragmentation patterns, see Table S1).

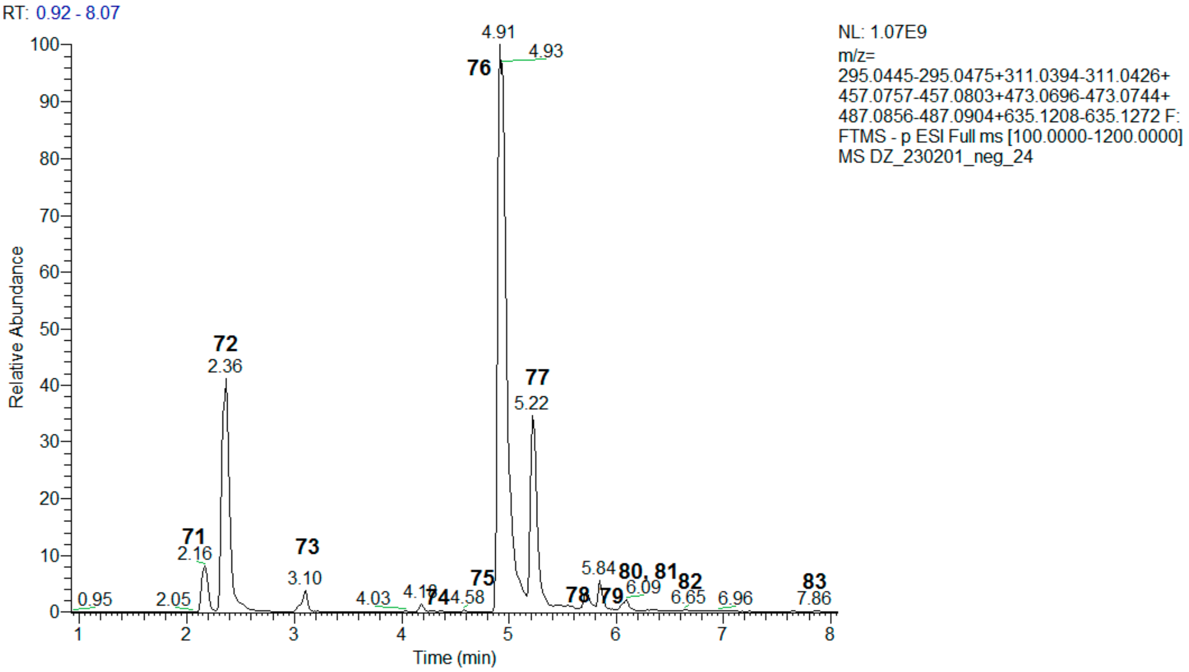

Figure S3. Extracted ion chromatograms of acyltartaric acids (for numbers and fragmentation patterns, see Table S1).

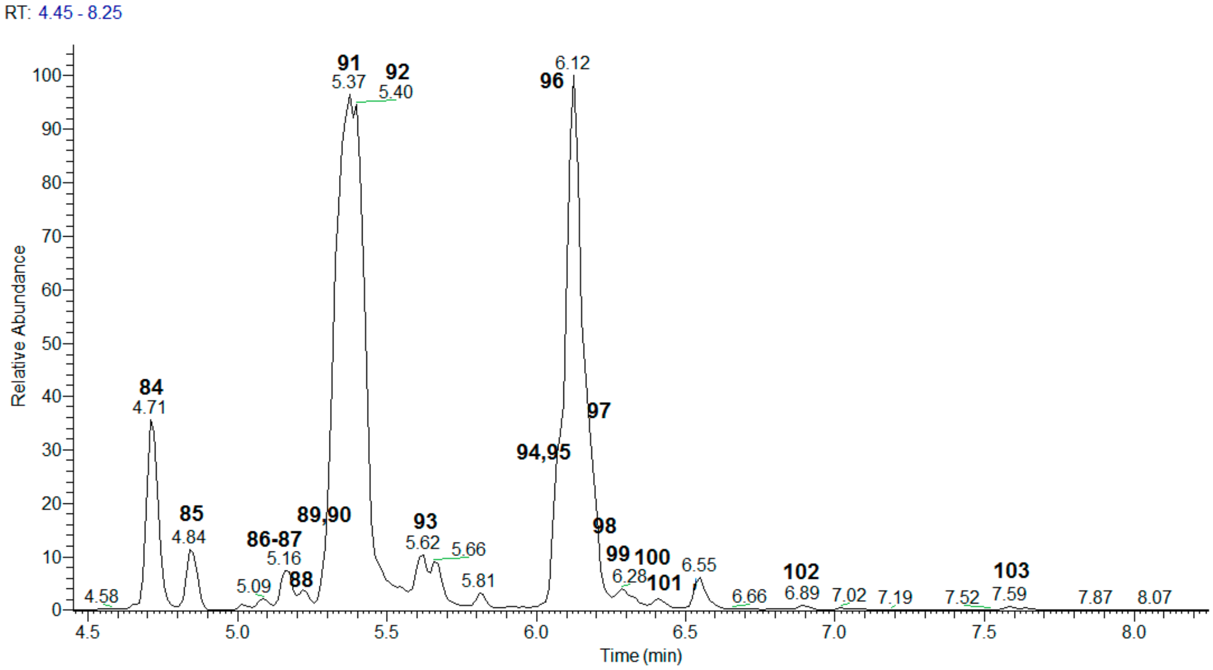

Figure S4. Extracted ion chromatograms of flavonoids (for numbers and fragmentation patterns, see Table S1).

References

[1] Sumner, L. W. et al. Proposed minimum reporting standards for chemical analysis: Chemical Analysis Working Group (CAWG) Metabolomics Standards Initiative (MSI). Metabolomics 2007, 3, p. 211–221.
